# Periodontitis as a Model for Inflammatory Uncoupling of Bone Remodeling

**DOI:** 10.1111/jre.70148

**Published:** 2026-07-06

**Authors:** Rafael Scaf de Molon, Sotirios Tetradis, Rolando Vernal, Fabio Renato Manzolli Leite, Thomas E. Van Dyke

**Affiliations:** ^1^ Department of Diagnostic and Surgery, School of Dentistry São Paulo State University (UNESP) Araçatuba Brazil; ^2^ Division of Diagnostic and Surgical Sciences UCLA School of Dentistry Los Angeles California USA; ^3^ Periodontal Biology Laboratory, Department of Conservative Dentistry, Faculty of Dentistry Universidad de Chile Santiago Chile; ^4^ Section of Population Health University of Utah School of Dentistry Salt Lake City Utah USA; ^5^ Center for Clinical and Translational Research ADA Forsyth Institute Somerville Massachusetts USA; ^6^ Department of Oral Medicine Infection and Immunity, Faculty of Medicine Harvard University Cambridge Massachusetts USA

**Keywords:** alveolar bone, bone, inflammatory disease, osteoclast, pathogenesis, periodontitis, signaling pathways

## Abstract

Periodontitis is traditionally regarded as an oral biofilm‐driven inflammatory disease that leads to progressive loss of the tooth‐supporting alveolar bone. However, accumulating evidence indicates that periodontal bone loss is more accurately understood as a state of pathological uncoupling of bone remodeling, in which exaggerated bone resorption coexists with inadequate bone formation response. In this review, we reposition periodontitis within the broader context of inflammatory skeletal diseases and synthesize current mechanistic insights from osteoimmunology, bone biology, and mechanobiology. We discuss how excessive osteoclastogenesis in periodontitis is sustained by receptor activator of nuclear factor kappa‐B ligand (RANKL) dominance derived from osteocytes, osteoblast‐lineage cells, stromal cells, monocytes/macrophages, B and T lymphocytes, and neutrophils within a cytokine‐rich microenvironment characterized by tumor necrosis factor (TNF)‐α, interleukin (IL)‐1β, IL‐6, and IL‐17A signaling. Persistent activation of nuclear factor kappa‐B (NF‐κB) and mitogen‐activated protein kinase (MAPK) pathways further enhance osteoclast differentiation, survival, and resorptive activity. At the same time, inflammatory mediators actively suppress osteoblast‐lineage commitment by inhibiting Runx2 and Osterix, antagonizing canonical Wnt/β‐catenin signaling through the upregulation of sclerostin and Dickkopf‐1 (DKK1), and impairing bone matrix production and mineralization. We further examine how disruption of key osteoclast–osteoblast coupling mechanisms, including ephrinB2/EphB4 and semaphorin signaling, prevents the effective transition from resorption to formation, while osteocyte dysfunction amplifies the uncoupled phenotype by integrating inflammatory and mechanical signals. Comparisons with rheumatoid arthritis, inflammatory bowel disease‐associated bone loss, and peri‐implantitis reveal shared immune‐driven mechanisms of remodeling imbalance, whereas the unique features of alveolar bone, including high turnover, continuous mechanical loading, and chronic microbial exposure, make it particularly susceptible to inflammatory uncoupling. Together, these concepts support a therapeutic shift toward restoring physiological coupling instead of solely inhibiting resorption and position periodontitis as a clinically accessible model for understanding and targeting inflammatory bone loss across skeletal diseases.

## Graphical abstract

Periodontitis can be viewed as a state of inflammatory uncoupling of bone remodeling, in which excessive osteoclast‐mediated resorption coexists with impaired bone formation. This review integrates emerging insights from osteoimmunology, osteocyte biology, mechanobiology, aging, and resolution pathways to position periodontitis as an accessible in vivo model of inflammation‐driven remodeling failure.

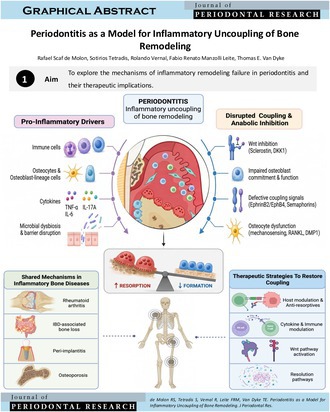

## 1 Introduction

Periodontitis is a highly prevalent chronic inflammatory disease initiated by dysbiotic oral biofilms and perpetuated by a dysregulated host response that promotes biofilm dysbiosis and progressively destroys the periodontal attachment tissues and the tooth‐supporting alveolar bone [1]. It represents a major global public health challenge. Recent Global Burden of Disease‐based estimates indicate that severe periodontitis affected more than 1 billion individuals worldwide in 2021, highlighting its substantial epidemiological impact and the urgent need for mechanism‐based preventive and therapeutic strategies [2, 3].

Beyond its local clinical sequelae, including periodontal pocket formation, tooth mobility, and eventual tooth loss, periodontitis has increasingly been recognized as a systemic inflammatory condition associated with the progression and severity of multiple chronic diseases, including type 2 diabetes, cardiovascular disease, osteoporosis, chronic kidney disease, rheumatoid arthritis, Alzheimer's disease, bacterial pneumonia, preeclampsia, inflammatory bowel disease, metabolic‐associated steatotic liver disease, and adverse pregnancy outcomes such as preterm birth and low birth weight [4, 5, 6, 7, 8, 9, 10, 11, 12, 13]. Importantly, the current staging and grading system recognizes that periodontitis reflects not only cumulative tissue destruction but also marked heterogeneity in disease behavior, susceptibility, and progression, thereby supporting the development of more precise and biologically informed approaches to risk stratification and host modulation [14, 15, 16].

Although these systemic associations underscore the broad inflammatory burden of periodontitis, the hallmark pathological feature responsible for tooth loss remains the progressive destruction of the periodontal supporting tissues. From an osteoimmunological perspective, inflammatory alveolar bone loss represents the defining lesion of periodontitis [17]. While traditionally viewed as a predominantly osteoclast‐driven process, increasing evidence indicates that periodontal bone loss reflects a broader remodeling imbalance characterized by enhanced bone resorption and insufficient osteoblast‐mediated repair [18, 19, 20, 21, 22]. This perspective has provided a basis for understanding disease progression beyond a purely resorptive model [23, 24]. In this context, Graves and colleagues elegantly proposed that inflammatory bone loss results not only from excessive osteoclast activity but also from impaired osteoblast‐mediated bone formation, introducing the concept of inflammation‐induced uncoupling as a fundamental mechanism of periodontal destruction [22]. This concept emphasizes that chronic inflammation disrupts the physiological coordination between bone resorption and bone formation that is required to maintain skeletal homeostasis.

Subsequent investigations have substantially expanded this basis by identifying additional cellular and molecular regulators that contribute to remodeling uncoupling. These include osteocytes, now recognized as central orchestrators of bone remodeling, Wnt signaling antagonists such as sclerostin and Dickkopf‐1, inflammatory signaling pathways, and mechanisms governing tissue resolution and regeneration [21, 22, 25, 26, 27, 28, 29]. More recently, the concept of uncoupling has been further refined through studies demonstrating how inflammatory networks, particularly NF‐κB‐dependent pathways, simultaneously promote osteoclastogenesis while suppressing osteoblast differentiation and function in periodontitis and other inflammatory skeletal disorders [25]. Collectively, these findings support the broader concept that chronic inflammation disrupts the molecular and cellular mechanisms responsible for coordinating physiological bone remodeling, thereby promoting persistent skeletal deterioration [17, 22, 25].

Building upon these advances, recent developments in resolution pharmacology, mechanobiology, aging research, and skeletal immunology have provided additional mechanistic insights into how inflammatory environments impair the restoration of bone homeostasis [30, 31, 32, 33, 34, 35, 36]. The present review integrates these emerging fields into a unified conceptual background that expands the theory of inflammatory uncoupling and explores its implications for understanding periodontitis as a model of inflammatory remodeling failure. Specifically, we discuss recent advances in osteocyte biology, sclerostin, Dickkopf‐1, and Wnt signaling, as well as emerging coupling pathways, such as ephrinB2/EphB4 and semaphorin signaling, which have significantly broadened current understanding of how inflammation disrupts remodeling homeostasis. These concepts may also have relevance to other inflammatory skeletal conditions characterized by remodeling imbalance, including rheumatoid arthritis, osteoporosis, and impaired fracture healing.

Although periodontitis shares numerous pathogenic mechanisms with other inflammatory skeletal disorders, including excessive osteoclastogenesis, suppression of osteoblast function, and disruption of physiological remodeling coupling, it arises within a unique biological context. Unlike osteoporosis, which is largely driven by systemic alterations in skeletal metabolism, or rheumatoid arthritis, which develops primarily within sterile synovial tissues, periodontitis occurs at a mucosal interface continuously exposed to a complex and dynamic microbial ecosystem [37, 38, 39]. Disease initiation and progression are closely linked to microbial dysbiosis, persistent activation of innate and adaptive immune pathways, and impairment of epithelial barrier integrity [38, 39, 40]. Consequently, inflammatory uncoupling in periodontitis emerges not only from dysregulated host inflammatory responses but also from sustained host–microbe interactions that perpetuate tissue damage and interfere with regenerative processes [21, 22]. These characteristics distinguish periodontitis from other inflammatory bone diseases while simultaneously providing a valuable opportunity to investigate the mechanisms through which chronic inflammation disrupts skeletal homeostasis in humans [41, 42, 43].

It is important to acknowledge that the application of remodeling concepts to inflammatory bone diseases remains a matter of ongoing discussion. Physiological bone remodeling is fundamentally a homeostatic process that preserves skeletal architecture through the coordinated balance of bone resorption and bone formation. In contrast, diseases such as periodontitis and rheumatoid arthritis are characterized by progressive tissue destruction and structural deterioration. Accordingly, the perspective proposed in this review does not suggest that inflammatory osteolysis represents physiological remodeling. Rather, it emphasizes that inflammatory bone loss develops, at least in part, through disruption of the cellular and molecular mechanisms that normally coordinate remodeling events, resulting in a persistent imbalance between resorption and formation.

Although previous reviews have explored inflammatory bone loss in periodontitis from the perspectives of osteoimmunology, host–microbe interactions, and signaling pathways [17, 21, 22], the present review adopts a distinct conceptual focus. We first examine the physiological basis of osteoclast–osteoblast coupling and then discuss how chronic periodontal inflammation disrupts this process through excessive bone resorption, suppression of bone formation, failure of coupling signals, and dysregulated immune‐mediated remodeling. We further integrate these mechanisms with emerging concepts from osteoimmunology, mechanobiology, aging biology, resolution pharmacology, and other inflammatory skeletal diseases to highlight the broader implications of periodontal bone loss for skeletal biology. By framing periodontitis through the lens of remodeling uncoupling, we aim to provide a mechanistic foundation for therapeutic strategies that not only suppress pathological resorption but also restore physiological coupling and alveolar bone homeostasis.

## 2 Physiological Coupling of Bone Remodeling: A Brief Overview

**FIGURE 1:**
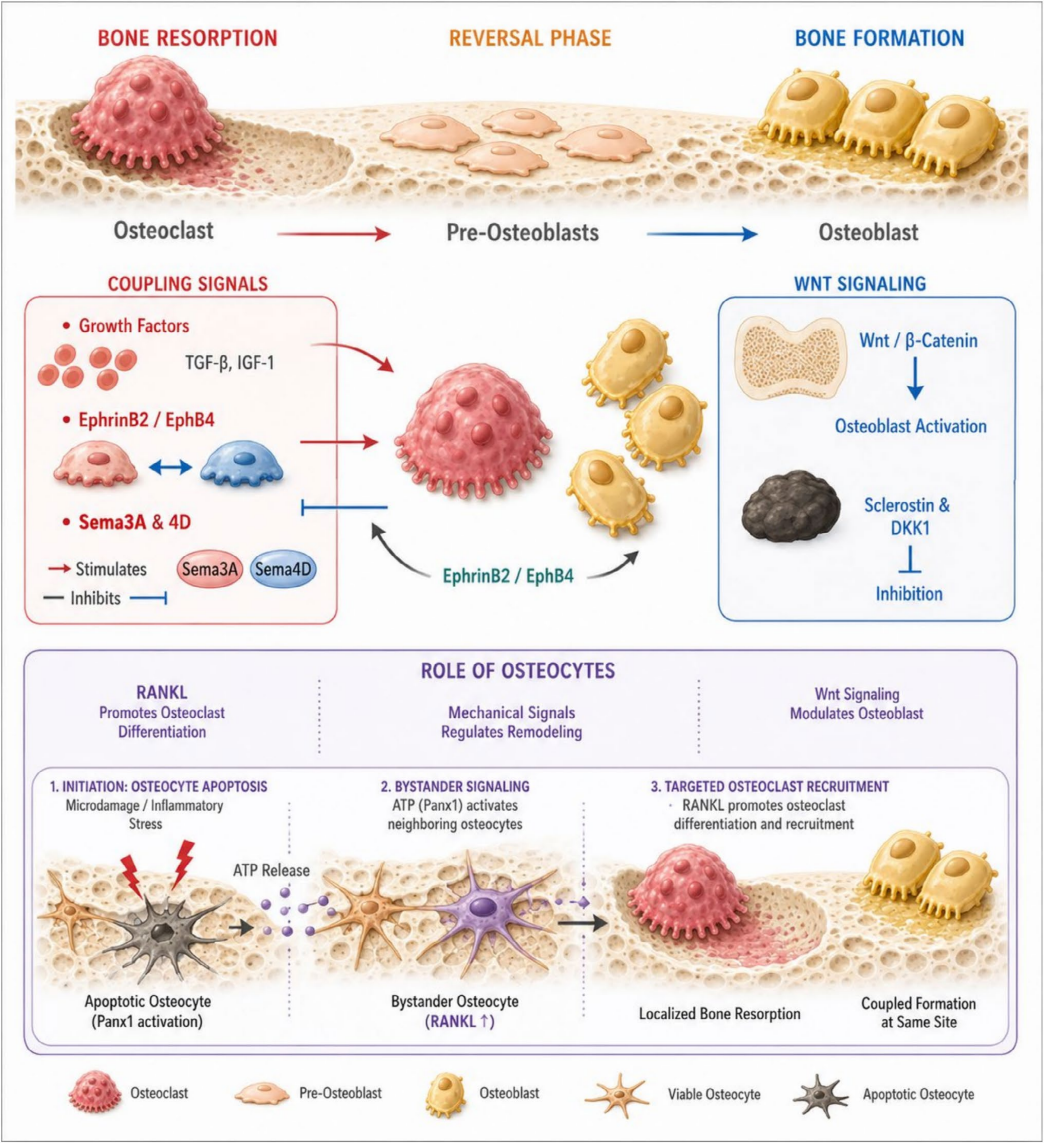
Cellular and molecular mechanisms governing coupled bone remodeling. Schematic representation of the classical bone remodeling cycle, illustrating the sequential phases of bone resorption, reversal, and bone formation. Osteoclasts initiate remodeling by resorbing the mineralized matrix. During the reversal phase, signals generated at the resorption site promote the recruitment and differentiation of pre‐osteoblasts, leading to osteoblast‐mediated osteoid deposition and subsequent mineralization. Osteoclast‐derived coupling signals, including growth factors such as TGF‐β and IGF‐1 released from the bone matrix, ephrinB2/EphB4 bidirectional signaling, and semaphorin‐3A/4D, coordinate communication between resorbing and bone‐forming cells. Canonical Wnt/β‐catenin signaling promotes osteoblast activation, whereas inhibitors such as sclerostin and DKK1 suppress bone formation. Osteocytes regulate remodeling initiation and spatial targeting through apoptosis‐dependent bystander signaling mechanisms. Osteocyte apoptosis induced by microdamage or inflammatory stress promotes ATP release through pannexin‐1 (Panx1) channels, stimulating neighboring viable osteocytes to upregulate RANKL and recruit osteoclast precursors to specific remodeling sites. In parallel, viable osteocytes regulate osteoblast activity through Wnt signaling and its antagonists, including sclerostin and DKK1. Together, these coordinated interactions maintain skeletal homeostasis by tightly coupling bone resorption to bone formation. DKK1, Dickkopf‐1; IGF‐1, insulin‐like growth factor‐1; RANKL, receptor activator of nuclear factor kappa B ligand; TGF, transforming growth factor. Created with BioRender.com (license no. WP29EC1XIK).

Physiological bone remodeling is a tightly coordinated process in which osteoclast‐mediated bone resorption is followed by osteoblast‐driven bone formation (Figure 1). This sequence is maintained through coupling mechanisms within the basic multicellular unit (BMU), involving reciprocal interactions among osteoclasts, osteoblast‐lineage cells, osteocytes, and the local immune microenvironment [44, 45]. Under homeostatic conditions, these coordinated interactions preserve bone mass and microarchitecture despite continuous skeletal turnover [45, 46].

The classical remodeling cycle begins with osteoclast differentiation, activation, and resorptive activity, followed by a reversal phase during which signals generated at the resorption site recruit and instruct osteoblast precursors [44, 45]. Osteoblasts subsequently deposit osteoid and regulate its mineralization, thereby completing the remodeling cycle. Importantly, osteoclasts are not merely bone‐resorbing cells; they also contribute to the initiation of bone formation by generating signals that promote osteoblast lineage commitment [47, 48, 49, 50]. This coupling concept is fundamental to skeletal homeostasis and provides a conceptual basis for understanding how remodeling disturbances lead to net bone loss [47, 48, 49, 50].

Multiple classes of coupling signals contribute to this coordination. One major mechanism involves the release of growth factors embedded within the bone matrix during osteoclastic resorption. Transforming growth factor (TGF)‐β and insulin‐like growth factor‐1 (IGF‐1) are among the best‐characterized examples [51]. Once released, these factors act on mesenchymal progenitors and osteoblast precursors to promote their recruitment, proliferation, and differentiation at sites of prior resorption, thereby linking anabolic activity to the extent of bone removal [51].

A fundamental but often overlooked feature of physiological bone remodeling is its spatial precision. Bone resorption is not initiated randomly but is selectively targeted to sites of microdamage or cellular stress, thereby preserving overall skeletal integrity. This targeting process is mediated, in part, through a bystander signaling mechanism initiated by apoptotic osteocytes. Following osteocyte death, pannexin‐1 (Panx1) membrane channels open and release adenosine triphosphate (ATP) into the extracellular environment. Extracellular ATP subsequently activates P2X7 receptors on neighboring viable osteocytes, inducing local RANKL expression and promoting the recruitment of osteoclast precursors to sites requiring repair [52, 53]. Genetic ablation of Panx1 prevents bystander RANKL induction and abolishes intracortical remodeling at sites of microdamage, demonstrating that the Panx1/ATP signaling axis is essential for the spatial coordination of remodeling initiation [52].

Through this mechanism, osteocytes function not only as mechanosensors but also as regulators that direct bone resorption to areas of genuine structural compromise, thereby ensuring that bone removal remains coupled to subsequent repair. These observations have important implications for inflammatory bone diseases. In periodontitis, osteocyte apoptosis is increasingly recognized as more than a passive consequence of tissue injury and may contribute to the initiation of osteoclastogenic signaling within affected alveolar bone [54, 55, 56, 57]. However, unlike the focal osteocyte death observed during physiological remodeling, chronic inflammatory conditions are associated with more widespread osteocyte apoptosis, potentially disrupting the spatial fidelity of remodeling and promoting diffuse bone resorption. Although the Panx1/ATP/P2X7‐dependent bystander mechanism linking apoptotic osteocytes to RANKL induction is well supported in bone microdamage and skeletal remodeling models [52, 53, 58, 59], its direct contribution to periodontal bone loss remains to be fully established.

An apparent paradox in inflammatory bone loss is that osteocyte apoptosis reduces the number of viable osteocytes capable of producing sclerostin, whereas elevated sclerostin expression is frequently reported in periodontitis and other inflammatory skeletal disorders [60, 61, 62, 63, 64]. This observation suggests that osteocyte death and osteocytic dysfunction do not occur uniformly throughout affected bone and that surviving osteocytes may undergo distinct phenotypic adaptations in response to inflammatory stimuli. Although the mechanisms underlying this phenomenon remain incompletely understood, emerging evidence indicates that inflammatory signaling may differentially regulate osteocyte survival and function [62, 65, 66]. The biological significance of this apparent paradox and its implications for remodeling uncoupling are discussed in later sections (Section 6.3).

Beyond soluble coupling factors, direct cell–cell communication within the BMU provides an additional layer of regulation that helps synchronize bone resorption and formation. Among the best‐characterized mechanisms is ephrinB2/EphB4 bidirectional signaling, which facilitates communication between osteoclasts and osteoblast‐lineage cells and contributes to the transition from resorption to subsequent bone formation [45]. Semaphorin signaling further refines these interactions by modulating the balance between osteoclastogenic and osteogenic responses, thereby supporting coordinated remodeling [67, 68]. Together, these contact‐dependent pathways demonstrate that osteoclasts actively participate in regulating the formation phase of remodeling.

The successful completion of this transition also depends on signaling pathways that promote osteoblast differentiation and anabolic activity. Among these, canonical Wnt/β‐catenin signaling plays a central role in supporting bone formation and maintaining remodeling balance. Its activity is tightly regulated by antagonists such as sclerostin and Dickkopf‐1 (DKK1), ensuring that anabolic responses are appropriately coupled to preceding resorptive events [46, 64, 69, 70, 71]. Integrating these regulatory networks are osteocytes, which function as the principal coordinators of skeletal homeostasis. Through their ability to sense mechanical and biological cues and regulate the production of factors that influence both osteoclasts and osteoblasts, osteocytes help orchestrate the spatial and temporal organization of remodeling within the bone remodeling unit [58, 59].

Collectively, these mechanisms establish the physiological basis that maintains skeletal integrity under normal conditions. Bone homeostasis depends not simply on controlling osteoclast or osteoblast activity in isolation, but on preserving the signaling networks that couple these processes in a coordinated manner. This physiological paradigm provides an essential reference for understanding how chronic inflammation disrupts remodeling coupling in periodontitis, ultimately leading to persistent alveolar bone loss.

## 3 Reframing Periodontitis as a Bone Remodeling Disorder

### 3.1 Why Periodontitis Is a Useful Model

The physiological processes that coordinate bone resorption and bone formation can become disrupted under chronic inflammatory conditions, resulting in progressive skeletal deterioration [72, 73, 74, 75, 76, 77]. Within this context, periodontitis represents a prototypical osteoimmunological disease in which persistent inflammation interferes with the mechanisms that normally maintain alveolar bone homeostasis [17, 18, 19, 20, 21, 22, 25, 72, 75, 78, 79, 80, 81]. Although periodontal bone destruction has traditionally been viewed as a predominantly osteoclast‐driven process, a resorption‐centered perspective does not fully explain the extent and persistence of tissue loss observed in susceptible individuals [18, 22, 25]. Rather, periodontitis can be understood as a disorder of remodeling imbalance in which bone resorption is inadequately followed by repair, resulting in progressive net bone loss.

This concept is not unique to periodontitis. Similar patterns of remodeling disruption have been described in other inflammatory skeletal diseases. In rheumatoid arthritis, focal bone erosions arise from sustained inflammatory osteoclast activation and are frequently accompanied by ineffective repair of the resulting defects, even when synovial inflammation is partially controlled [43, 82]. Likewise, peri‐implantitis exhibits aggressive inflammatory osteolysis with limited regenerative capacity, whereas chronic osteomyelitis is characterized by persistent bone destruction despite the inherent osteogenic potential of skeletal tissues [83, 84]. Collectively, these observations suggest that remodeling uncoupling may represent a common biological consequence of chronic inflammation across diverse skeletal conditions.

At the same time, periodontitis differs from these diseases in several important respects. Its unique anatomical location, exposure to a complex microbial environment, and dependence on continuous functional loading create a biological setting in which the mechanisms governing remodeling balance can be studied in a particularly dynamic and clinically accessible manner, as described below.

### 3.2 Mechanical Loading as a Modulator of Remodeling

Mechanically, unlike long bones, which primarily provide structural support and mineral homeostasis, alveolar bone must continuously remodel to accommodate changes in occlusion, mastication, and tooth position [82, 85, 86]. Forces generated during chewing are transmitted through the periodontal ligament to the alveolar bone, producing localized strain patterns that strongly influence bone cells. Osteocytes embedded within alveolar bone sense these mechanical signals and coordinate remodeling responses through mechanotransduction pathways, including Wnt/β‐catenin signaling and sclerostin regulation [1, 20, 22, 82]. As a result, alveolar bone exhibits a high basal remodeling rate even under physiological conditions [18]. Recent integrative views further suggest that inflammatory mediators can intersect with mechanotransduction pathways to distort bone turnover dynamics in the craniofacial complex, thereby influencing both periodontal breakdown and repair [87]. Thus, although the fundamental principles of remodeling coupling that govern other skeletal sites are likely also operative in the periodontium, they do so in the presence of site‐specific amplifiers that may favor persistent uncoupling.

### 3.3 Microbial Triggers and Chronic Immune Activation

A defining feature that further distinguishes alveolar bone from other skeletal sites is its immediate proximity to microbial biofilms. The periodontium forms a unique interface between mineralized tissues and a dense polymicrobial community residing on the tooth surface. In health, the subgingival biofilm exists in a state of dynamic equilibrium with the host immune response, with commensal microorganisms actively contributing to homeostasis [18, 19]. This equilibrium can be disrupted by inflammation induced by both local factors, such as inadequate oral hygiene and biofilm accumulation, and systemic factors including diabetes, tobacco smoking, psychological stress, hormonal changes, and immune dysregulation, all of which can alter the composition and virulence potential of the subgingival community [88, 89]. Once dysbiosis is established, the resulting microbial community generates a persistent source of microbial‐associated molecular patterns that penetrate adjacent tissues and drive local inflammation. Critically, this relationship is bidirectional: inflammatory tissue breakdown products serve as nutrients for the dysbiotic microbiota, so‐called inflammophiles, creating a self‐sustaining feedforward loop in which dysbiosis perpetuates inflammation and inflammation perpetuates dysbiosis [18, 19, 20, 21]. Unlike long bones, which are shielded from direct microbial exposure, alveolar bone is uniquely positioned at this host‐biofilm interface and is therefore chronically subjected to this self‐reinforcing inflammatory cycle [1, 20, 22]. This constant exposure creates conditions in which bone cells are repeatedly challenged by inflammatory mediators that interfere with normal remodeling processes.

Alveolar bone is also characterized by rich vascularization and pronounced immune cell infiltration, particularly within the periodontal ligament and adjacent marrow spaces [17]. This vascular network enables rapid recruitment of primary and secondary immune cells in response to microbial challenge, a feature essential for host defense [17]. However, sustained accumulation of immune cells leads to prolonged exposure of bone‐resident cells to cytokines such as tumor necrosis factor (TNF)‐α, interleukin (IL)‐1β, IL‐17A, and RANKL, which promote osteoclastogenesis while simultaneously suppressing osteoblast differentiation and function [1, 20, 22, 82, 90]. The intimate integration of the immune and skeletal compartments in the periodontium thus amplifies the impact of inflammation on remodeling balance.

### 3.4 Disease‐Specific Differences That Distinguish Periodontitis

The characteristics mentioned above underscore fundamental differences between alveolar bone and long bones or trabecular bone elsewhere in the skeleton. In axial and appendicular sites, remodeling occurs within a relatively insulated environment, with inflammatory insults often arising systemically rather than locally. Even in inflammatory skeletal diseases such as RA, bone loss typically reflects focal erosions adjacent to inflamed synovium instead of continuous exposure to microbial products [43]. In contrast, alveolar bone experiences the convergence of mechanical loading, microbial challenge, and immune activation within a confined anatomical space, predisposing it to more severe and persistent remodeling disturbances.

The implications for susceptibility to remodeling imbalance are substantial. The high turnover rate of alveolar bone means that disruption of coupling signals can rapidly translate into net bone loss [22]. Mechanical loading, which would normally promote bone formation, may fail to exert anabolic effects when inflammatory mediators blunt osteoblast responsiveness or enhance osteocyte‐derived inhibitors of bone formation [55, 80]. Persistent microbial stimulation and immune infiltration further reinforce a resorption‐dominated environment, preventing effective engagement of the formation phase. Consequently, the alveolar bone is a skeletal site where remodeling uncoupling is readily initiated and difficult to resolve, providing a biological explanation for the progressive and often irreversible nature of periodontal bone loss [17, 22, 80].

These site‐specific features position alveolar bone as a high‐turnover, inflammation‐prone tissue in which local environmental pressures can overwhelm physiological coupling, framing the mechanistic pathways discussed in the following sections.

It is worth noting that the rate and intensity of remodeling uncoupling in periodontitis are not uniform across patients or sites. The 2018 staging and grading classification explicitly encodes this heterogeneity, recognizing that some patients and sites experience periods of more rapid bone loss while others show relative stability [14, 15]. Within the uncoupling context, this reflects variation in the local balance between inflammatory drive, osteoclastogenic signaling, and the residual capacity for coupled formation, with the defining pathological feature being not the uninterrupted continuity of active resorption but the recurrent failure of effective repair whenever destructive episodes occur.

## 4 Exaggerated Bone Resorption in Periodontitis

### 4.1 The “Overactive” Arm of Periodontitis‐Associated Alveolar Bone Loss

A defining hallmark of periodontal bone loss is the persistent, localized activation of bone resorption. While osteoclast activity is essential for physiological remodeling, periodontitis drives excessive and sustained osteoclastogenesis, tipping the remodeling balance toward net bone loss [1]. This resorptive dominance is maintained by an inflammatory microenvironment that amplifies osteoclast differentiation, prolongs survival, and alters function, largely through dysregulated RANKL expression and increased precursor responsiveness to pro‐resorptive signals [17, 29]. Thus, exaggerated bone resorption represents the “overactive” arm of remodeling uncoupling in periodontitis.

### 4.2 Cellular Sources of RANKL

**FIGURE 2:**
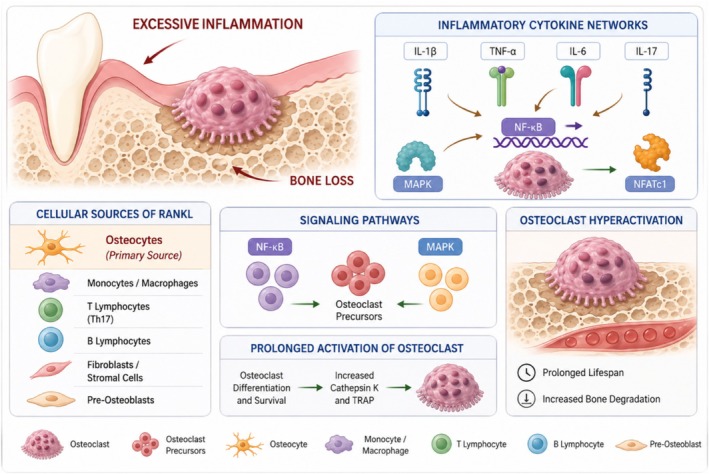
Inflammatory mechanisms drive osteoclast hyperactivation and alveolar bone loss during periodontitis. Schematic illustration of the cellular and molecular pathways underlying inflammation‐induced alveolar bone resorption during periodontitis. Excessive inflammatory stimulation within the periodontal microenvironment activates cytokine networks involving IL‐1β, TNF‐α, IL‐6, and IL‐17A. These mediators stimulate intracellular signaling cascades, including the NF‐κB, MAPK, and NFATc1 pathways, which promote osteoclast differentiation and functional activation. Multiple cellular sources contribute to RANKL production, including pre‐osteoblasts, fibroblasts, osteocytes, and adaptive immune cells, particularly Th17 and B cells, thereby amplifying osteoclastogenesis. Sustained signaling enhances osteoclast precursor recruitment, differentiation, and survival, while increasing the expression of resorptive enzymes such as TRAP and cathepsin K. The cumulative effect is osteoclast hyperactivation, prolonged resorptive activity, and progressive alveolar bone loss characteristic of periodontitis. MAPK, mitogen‐activated protein kinase; NFATc1, nuclear factor of activated T cells c1; NF‐κB, nuclear factor kappa B; RANKL, receptor activator of nuclear factor kappa B ligand; TRAP, tartrate‐resistant acid phosphatase. Created with BioRender.com (license no. ZH29ECIOJ8).

RANKL is the essential cytokine governing osteoclast differentiation, activation, and survival, and its availability within periodontal tissues is a critical determinant of resorptive activity (Figure 2). Although early models emphasized immune cells as the principal source of RANKL in inflammatory bone loss, accumulating evidence indicates that bone‐resident cells are dominant contributors in periodontitis [1].

Among these, osteocytes have emerged as the principal source of functionally relevant RANKL driving alveolar bone resorption [55, 59, 91, 92]. Genetic studies in mice have shown that selective deletion of RANKL from osteocytes, but not from osteoblasts, provides substantial protection against alveolar bone loss in experimental periodontitis, establishing osteocytes as indispensable regulators of osteoclastogenesis [54]. This paradigm mirrors findings at other skeletal sites, where osteocyte‐derived RANKL is now recognized as a dominant driver of both physiological and pathological bone resorption [93, 94].

Osteoblast‐lineage cells, including pre‐osteoblasts and stromal progenitors, also contribute to the RANKL pool in periodontal tissues. Inflammatory cytokines markedly increase RANKL expression in these cells while suppressing osteoprotegerin (OPG), the soluble decoy receptor for RANKL, thus increasing the RANKL/OPG ratio and favoring osteoclastogenesis [22, 95]. This shift is particularly relevant in the inflamed periodontium, where osteogenic cells are exposed to sustained cytokine signaling.

In addition to skeletal cells, activated T and B lymphocytes represent important immunological sources of RANKL in periodontitis. B cells, which accumulate in advanced periodontal lesions, express RANKL and contribute to local osteoclastogenic signaling, particularly under chronic inflammatory conditions [96, 97]. Gingival fibroblasts and other stromal cells exposed to microbial products and inflammatory cytokines likewise upregulate RANKL expression, further expanding the cellular network that supports osteoclastogenesis in periodontitis‐affected tissues [98]. Inflammatory macrophages and monocytes represent an additional cellular source of pro‐osteoclastogenic signals in the periodontitis microenvironment. Although their direct RANKL‐expressing capacity varies with activation state and context, inflammatory macrophages contribute to osteoclastogenesis primarily through the sustained production of TNF‐α, IL‐1β, and IL‐6, which amplify osteoclast precursor differentiation and survival, and through NF‐κB‐dependent inflammatory circuits that enhance cellular responsiveness to RANKL signaling [67, 68, 69, 70, 71, 72].

Macrophage effects on osteoclastogenesis are phenotype‐ and context‐dependent: classically activated macrophages can also secrete IFN‐γ and IL‐12, which may restrain osteoclast formation under certain conditions [99, 100, 101], yet the net effect in the chronic periodontal lesion is pro‐resorptive, reflecting the dominance of TNF‐α‐driven signaling over homeostatic counter‐regulation [102, 103, 104, 105]. The myeloid contribution to both arms of remodeling uncoupling is discussed further in Section 7.5.

### 4.3 Pro‐Inflammatory Cytokine Networks

The exaggerated expression of RANKL in periodontitis is tightly linked to a pro‐inflammatory cytokine milieu dominated by TNF‐α, IL‐1β, IL‐6, and IL‐17A. These cytokines act both upstream and downstream of RANKL to amplify osteoclastogenic signaling [17]. TNF‐α and IL‐1β directly promote osteoclast precursor differentiation and synergize with RANKL to enhance osteoclast formation and activity [17]. Importantly, TNF‐α can drive osteoclastogenesis even under conditions of limited RANKL availability, thereby lowering the threshold for resorptive activation [43, 106]. IL‐6, particularly through trans‐signaling mechanisms, further supports osteoclast differentiation and survival in inflammatory settings [107]. IL‐17A, a key Th17 cytokine, promotes periodontal bone loss by inducing RANKL expression in osteoblast‐lineage and stromal cells while increasing osteoclast precursor sensitivity to RANK signaling [108, 109, 110]. Th17 cells also express both membrane‐bound and soluble RANKL and contribute to osteoclast differentiation in periodontal lesions [108, 109, 110]. This establishes a direct link between adaptive immunity and skeletal destruction.

At the intracellular level, these pro‐inflammatory mediators converge on the NF‐κB and mitogen‐activated protein kinase (MAPK) pathways, which constitute the core transcriptional machinery governing osteoclast differentiation, activation, and survival [17, 29, 111]. Activation of these pathways downstream of RANK, TNF receptors, and IL‐1 receptors leads to the rapid induction of osteoclastogenic transcription factors, most notably nuclear factor of activated T cells c1 (NFATc1), the master regulator of osteoclast lineage commitment [17, 29, 111]. NF‐κB signaling initiates NFATc1 expression, whereas mitogen‐activated protein kinase (MAPK) cascades, including extracellular signal‐regulated kinase (ERK), p38, and c‐Jun N‐terminal kinase (JNK), stabilize and amplify this program by promoting transcriptional activity and sustaining osteoclast precursor responsiveness [17, 29, 111].

In periodontal tissues, these signaling pathways are not transiently engaged but persistently activated by continuous exposure to microbial stimuli and inflammatory cytokines [17]. This prolonged signaling environment reinforces osteoclastogenic gene expression far beyond the physiological window of remodeling activation, thereby favoring excessive osteoclast differentiation and enhanced resorptive function [17]. Sustained NF‐κB and MAPK activity also delays osteoclast apoptosis, extending osteoclast lifespan and increasing cumulative bone resorption at affected sites [17]. This persistence contrasts sharply with acute inflammatory responses, in which activation of these pathways is tightly regulated and resolves once the inciting stimulus is removed.

Accordingly, chronic activation of NF‐κB‐ and MAPK‐dependent transcriptional pathways represents a major molecular distinction between periodontitis and transient inflammatory insults. Functioning as short‐lived mediators of host defense, these pathways become central drivers of pathological bone loss, locking the remodeling system into a resorption‐dominant state. This sustained intracellular signaling provides a mechanistic explanation for the progressive and often irreversible nature of alveolar bone destruction in periodontitis and aligns periodontal bone loss with other chronic inflammatory osteolytic diseases [29, 111].

### 4.4 Osteoclast Hyperactivation

The combined actions of expanded RANKL availability and sustained cytokine signaling culminate in osteoclast hyperactivation. Histological and experimental studies consistently demonstrate increased osteoclast numbers along alveolar bone surfaces in periodontitis, accompanied by enlarged resorption lacunae and increased expression of osteoclast functional markers such as cathepsin K and tartrate‐resistant acid phosphatase (TRAP) [22, 72, 75, 112, 113, 114, 115].

Beyond increased osteoclast numbers, inflammatory environments also confer a distinct functional phenotype. Pro‐survival signals driven by TNF‐α and RANKL delay osteoclast apoptosis, whereas inflammatory mediators enhance resorptive capacity at the single‐cell level [116]. Emerging evidence further suggests that inflammatory osteoclasts differ transcriptionally and metabolically from osteoclasts generated during physiological remodeling, displaying heightened responsiveness to inflammatory signals and greater bone‐degrading activity [117]. These features closely parallel observations in RA and other forms of inflammatory osteolysis, where excessive osteoclast activation at erosion sites drives progressive bone destruction. Collectively, these findings establish exaggerated bone resorption as a central pathological component of periodontitis. However, as discussed in the following section, excessive resorption alone does not fully explain persistent alveolar bone loss.

## 5 Suppressed Bone Formation in Periodontitis

### 5.1 The “Underestimated” Side of Periodontitis‐Associated Alveolar Bone Loss

While increased osteoclast activity is a well‐recognized feature of periodontal bone loss, accumulating evidence shows that impaired bone formation is an equally critical and distinct contributor [22, 118]. Importantly, this suppression is not constitutive but dynamically regulated: experimental models demonstrate that early or mild inflammatory states may initially elicit compensatory osteogenic responses, whereas sustained chronic inflammation progressively dismantles osteoblast‐lineage commitment and function [119]. The net suppression of bone formation characteristic of established periodontitis therefore reflects a stage‐dependent and inflammation‐intensity‐dependent failure of the coupling response.

### 5.2 Inhibition of Osteoblast Differentiation

Chronic periodontal inflammation directly interferes with osteoblast differentiation by targeting key transcriptional regulators of osteogenesis. TNF‐α and IL‐1β, both abundantly expressed in periodontal lesions, suppress the expression and activity of Runx2 and Osterix (Sp7), two master transcription factors required for osteoblast lineage commitment and maturation [120, 121]. Persistent exposure to TNF‐α not only reduces Runx2 transcriptional activity but also promotes its post‐translational destabilization, impairing its ability to coordinate downstream osteogenic gene expression [120, 121]. In parallel, IL‐1β exerts inhibitory effects particularly during early differentiation stages, reinforcing a blockade that prevents progenitor cells from acquiring an osteoblastic phenotype [122]. Importantly, these effects are not transient; sustained cytokine exposure establishes a stable inhibitory state that prevents effective recovery of osteogenic potential even when local conditions fluctuate [120, 121].

At the molecular level, these cytokines act largely through NF‐κB‐dependent mechanisms that antagonize osteogenic gene expression. Activation of NF‐κB signaling in osteoblast precursors represses Runx2‐ and Osterix‐driven transcription and downregulates genes encoding bone matrix proteins, including type I collagen and osteocalcin [119]. Importantly, NF‐κB activation not only delays osteoblast differentiation but may also redirect progenitor cells toward alternative, non‐osteogenic lineages, thereby reducing the pool of cells available for bone formation [119]. Within periodontal lesions, osteoblast lineage commitment becomes progressively inefficient, and the normal replenishment of osteoblasts required to complete the remodeling cycle is compromised. This failure to generate sufficient numbers of functional osteoblasts represents a critical early event in the suppression of bone formation.

### 5.3 Wnt Signaling Antagonism

Beyond directly suppressing osteoblast differentiation, periodontal inflammation disrupts bone formation by antagonizing canonical Wnt/β‐catenin signaling, a pathway essential for osteoblast proliferation, differentiation, survival, and anabolic activity [69]. Unlike direct cytokine‐mediated inhibition of transcription factors, Wnt antagonism operates at the level of extracellular signaling control, effectively silencing anabolic pathways even in the presence of osteogenic stimuli. Clinical and experimental studies report increased expression of Wnt inhibitors, most notably sclerostin and DKK1, in periodontal tissues and in the systemic circulation of individuals with periodontitis [123]. These antagonists act by binding to LRP5/6 co‐receptors, thereby preventing Wnt ligands from activating β‐catenin‐dependent transcriptional factors [124, 125]. The result is a broad suppression of osteoblast proliferation, differentiation, and survival, limiting both the expansion and functional activity of osteoblast‐lineage cells. Accordingly, cytokines such as TNF‐α and IL‐1β upregulate sclerostin expression, thereby directly linking inflammation to suppression of bone formation [126, 127]. Similarly, DKK1 is induced by pro‐inflammatory cytokines and has been implicated in impaired osteoblast activity in inflammatory skeletal diseases, including RA and periodontitis [128, 129]. As a consequence of increased Wnt antagonism, β‐catenin signaling is reduced in osteoblast precursors within periodontal lesions, leading to decreased osteoblast survival and diminished anabolic responses [55, 91, 130, 131]. Importantly, emerging evidence suggests that resolution‐phase mediators may indirectly restore Wnt/β‐catenin signaling by suppressing inflammatory pathways responsible for induction of sclerostin and DKK1, thereby linking resolution biology to restoration of anabolic remodeling [27, 126, 127, 129, 132, 133, 134, 135, 136, 137].

### 5.4 Impaired Matrix Production and Mineralization

Even when osteoblasts are present within periodontitis‐affected tissues, their functional capacity to produce and mineralize the extracellular matrix is somehow compromised. Chronic inflammation reduces type I collagen synthesis, the principal structural component of bone matrix, through cytokine‐mediated repression of collagen gene expression and increased matrix degradation through proteolytic mechanisms [138, 139]. This reduction in matrix production limits the substrate available for subsequent mineralization and weakens newly formed bone. Inflammatory conditions also alter alkaline phosphatase (ALP) activity, a key enzyme required for hydroxyapatite deposition and mineral maturation [122]. Experimental models have shown that TNF‐α and IL‐1β suppress ALP expression and activity in osteoblasts, resulting in defective mineralization even in the presence of osteogenic stimuli [122]. These effects further decouple matrix deposition from mineral incorporation, leading to structurally inferior bone.

In addition, oxidative stress, a hallmark of chronic periodontal inflammation, directly impairs osteoblast function [140, 141, 142]. Elevated levels of reactive oxygen species inhibit osteoblast differentiation, reduce matrix protein synthesis, and promote osteoblast apoptosis [143]. Oxidative stress also disrupts Wnt/β‐catenin signaling, thereby further suppressing anabolic pathways [144]. Collectively, bone formation remains inefficient and poorly coordinated, even when resorption has created remodeling surfaces. By highlighting the molecular and cellular mechanisms that restrain osteogenesis, this section underscores the need to consider impaired bone formation a consequence of disease, but also a therapeutic target in its own right.

Although accumulating evidence supports the concept that inflammatory suppression of bone formation contributes to periodontal bone loss, the strength of evidence remains more heterogeneous than that supporting osteoclast‐mediated resorption. Consequently, several mechanisms discussed above should be interpreted as emerging components of the anabolic arm of remodeling dysregulation and not as fully established drivers of periodontal pathology.

## 6 Disruption of Osteoclast–Osteoblast Coupling Signals

### 6.1 The Core of the Osteoclast–Osteoblast Uncoupling During Periodontitis

The central feature of osteoclast–osteoblast uncoupling in periodontitis lies in the disruption of signaling pathways that normally coordinate sequential bone resorption and formation. These coupling mechanisms ensure spatially and temporally regulated remodeling with effective anabolic responses. In periodontitis, however, this regulatory network is compromised, as described below.

### 6.2 Altered Coupling Factors

One of the best‐characterized coupling pathways is the ephrinB2/EphB4 axis, which mediates bidirectional signaling between osteoclasts and osteoblast‐lineage cells. Under physiological conditions, osteoclast‐expressed ephrinB2 interacts with EphB4 receptors on osteoblast precursors, promoting osteoblast differentiation while simultaneously restraining further osteoclast activity [145, 146]. Importantly, this pathway operates as a local, contact‐dependent regulatory system that links the presence of active osteoclasts to the recruitment and activation of osteoblasts at the same remodeling site. In this context, ephrinB2/EphB4 signaling functions as a molecular “handover” mechanism, ensuring that bone removed during resorption is subsequently replaced.

Disruption of this axis therefore has profound consequences, as it uncouples the spatial and temporal coordination between the two cell types. Impaired EphB4 signaling in osteoblast precursors limits their differentiation and responsiveness to resorptive signals, while reduced reverse signaling in osteoclasts prolongs their activity, collectively favoring a resorption‐dominant state [145, 146]. Although direct investigation of ephrin signaling in periodontitis remains limited, inflammatory cytokines that dominate periodontal lesions, particularly TNF‐α and IL‐1β, have been shown in other skeletal contexts to suppress EphB4 expression and interfere with ephrin‐mediated anabolic signaling [43, 146]. Given the sustained inflammatory environment of periodontal tissues, it is plausible that similar mechanisms contribute to defective coupling in alveolar bone, thereby preventing osteoblast‐lineage cells from responding appropriately to prior resorption [22]. As these mechanisms have not been directly demonstrated in periodontitis, further studies are warranted to demonstrate this possible mechanism in the alveolar bone.

A second major class of coupling mediators affected under inflammatory conditions is the semaphorin family. Originally characterized as axonal guidance molecules, semaphorins have emerged as critical regulators of bone remodeling by modulating cell–cell communication within the BMU. In particular, Sema3A and Sema4D exert opposing effects on bone formation and resorption, thereby contributing to the fine‐tuning of remodeling balance. Sema3A exerts anabolic effects by promoting osteoblast differentiation and inhibiting osteoclast formation [67, 68]. In contrast, Sema4D acts as a negative regulator of bone formation. By binding to its receptor Plexin‐B1 on osteoblast‐lineage cells, Sema4D inhibits osteoblast differentiation, reduces cytoskeletal organization, and suppresses matrix production. This signaling pathway provides a mechanism by which active osteoclasts can directly limit osteoblast activity, thereby modulating the extent and timing of bone formation. Under normal conditions, the balance between Sema3A and Sema4D signaling ensures that bone formation is initiated appropriately and remains tightly controlled. Evidence from RA and inflammatory osteolysis indicates that increased Sema4D expression by activated osteoclasts directly impairs osteoblast differentiation and matrix production [68].

Although semaphorin signaling pathways, particularly Sema3A and Sema4D, are well‐established regulators of osteoblast and osteoclast activity during physiological and pathological skeletal remodeling [45, 50, 67, 68, 145], their specific contribution to periodontal bone loss remains poorly defined. Likewise, direct evidence implicating ephrinB2/EphB4 signaling in periodontitis is currently limited. Nevertheless, studies in physiological bone remodeling, rheumatoid arthritis, and other inflammatory osteolytic conditions suggest that inflammatory environments can disrupt these coupling pathways, thereby impairing the transition from bone resorption to bone formation [43, 45, 50, 82, 145, 146, 147]. Consequently, the involvement of semaphorin‐ and ephrin‐mediated uncoupling in periodontitis should presently be regarded as biologically plausible but not yet definitively established.

In addition to disrupting coupling signals, chronic inflammation appears to alter the functional phenotype of osteoclasts themselves. Under inflammatory conditions, osteoclasts may retain high resorptive activity while exhibiting a reduced capacity to produce anabolic and coupling factors that normally promote osteoblast recruitment and differentiation [145]. This qualitative shift further compromises the initiation of bone formation following resorption and highlights an important distinction between physiological and pathological osteoclast activity.

Collectively, these observations suggest that remodeling uncoupling in inflammatory bone loss extends beyond excessive osteoclastogenesis and involves broader disruption of the signaling networks that coordinate communication between resorptive and formative cells. Accordingly, therapeutic strategies that suppress inflammation and promote resolution may contribute to the restoration of physiological coupling, at least in part, by preserving or re‐establishing these regulatory pathways.

### 6.3 Osteocyte‐Mediated Uncoupling

Beyond altered osteoclast–osteoblast communication, osteocytes play a central role in enforcing uncoupling in periodontitis [58, 59, 93, 148, 149]. As the most abundant bone cells and master regulators of remodeling, osteocytes integrate mechanical, inflammatory, and metabolic signals to coordinate both resorption and formation [56, 58, 62, 65]. During periodontitis, osteocyte viability and signaling capacity are compromised [55, 59, 91, 92]. Histological and experimental studies have documented osteocyte apoptosis and increased numbers of empty lacunae in alveolar bone exposed to chronic inflammation [55, 130]. Osteocyte death is a potent stimulus for osteoclast recruitment and activation, acting as a danger signal that promotes localized bone resorption [55, 130]. During periodontitis, osteocyte apoptosis therefore contributes to exaggerated resorption while simultaneously reducing the number of cells capable of coordinating anabolic responses [55, 130, 150].

Under inflammatory conditions, osteocytes exhibit disrupted lacunar–canalicular signaling, impairing mechanotransduction and long‐range communication with surface bone cells. This limits their ability to transmit anabolic signals in response to mechanical loading, thereby weakening osteoblast activation at sites of resorption [151, 152, 153, 154, 155]. Consequently, alveolar bone fails to mount an effective bone‐forming response even under functional loading [91, 156].

As introduced in the previous section, inflammatory bone loss presents an apparent contradiction in which elevated sclerostin expression coexists with increased osteocyte apoptosis. This observation is particularly intriguing because sclerostin, a potent inhibitor of Wnt/β‐catenin signaling and bone formation, is produced primarily by viable osteocytes [46, 157]. Accordingly, one might expect osteocyte loss to reduce local sclerostin levels. However, both periodontal tissues and the systemic circulation of individuals with periodontitis exhibit increased sclerostin expression [123, 150, 158].

Current evidence suggests that these seemingly opposing observations reflect distinct responses of osteocyte populations to inflammatory stimuli and not a direct causal relationship. TNF‐α, a central mediator of periodontal inflammation, has been shown to induce SOST transcription in surviving osteocytes, increasing both SOST mRNA expression in vitro and the proportion of sclerostin‐positive osteocytes in vivo [159]. Thus, chronic inflammation may simultaneously drive apoptosis in some osteocytes while promoting enhanced RANKL and sclerostin expression in neighboring viable cells through TNF‐α/NF‐κB‐dependent signaling pathways. In this model, osteocyte apoptosis and sclerostin upregulation are not mutually exclusive events but parallel consequences of a shared inflammatory environment.

Support for this concept is provided by studies in ligature‐induced periodontitis associated with diabetes, in which TNF‐α blockade with infliximab reduced the number of RANKL‐positive and sclerostin‐positive osteocytes while partially restoring osteoid formation [160, 161, 162, 163]. These findings suggest that inflammatory signaling not only promotes osteocyte‐mediated osteoclastogenesis but may also contribute to the suppression of bone formation through increased sclerostin production, thereby reinforcing remodeling uncoupling during periodontal bone loss.

Taken together, these mechanisms suggest a proposed integrative model in which osteocyte apoptosis, triggered by inflammatory cytokines, oxidative stress, and hypoxia within the periodontal lesion downstream of dysbiotic biofilm‐driven immune activation, functions as a proximal cellular trigger for targeted osteoclast recruitment at individual bone sites. Dying osteocytes release ATP through pannexin‐1 channels, signaling bystander viable osteocytes to upregulate RANKL and thereby initiating localized osteoclast recruitment [52]. Simultaneously, TNF‐α stimulates surviving osteocytes to upregulate both RANKL and sclerostin, amplifying the resorptive drive while suppressing anabolic Wnt signaling [160]. The chronic inflammatory microenvironment prevents effective engagement of the coupled formation phase by suppressing osteoblast‐lineage commitment, Runx2 activity, and Wnt/β‐catenin signaling. Further osteocyte death in the progressively exposed bone layer may initiate subsequent resorptive episodes, creating a self‐amplifying cycle. This feedforward model positions osteocyte biology at the center of periodontal bone loss, with the critical mechanistic distinction from physiological targeted remodeling lying not in the resorption‐initiation mechanism itself but in the failure of the subsequent repair response [54, 92, 131]. Direct experimental testing of this model in the periodontitis context remains an important priority for future research.

## 7 Osteoimmunology: Immune‐Driven Uncoupling of Remodeling

### 7.1 Cross‐Talk Between Immune Cells and Bone Cells

**FIGURE 3:**
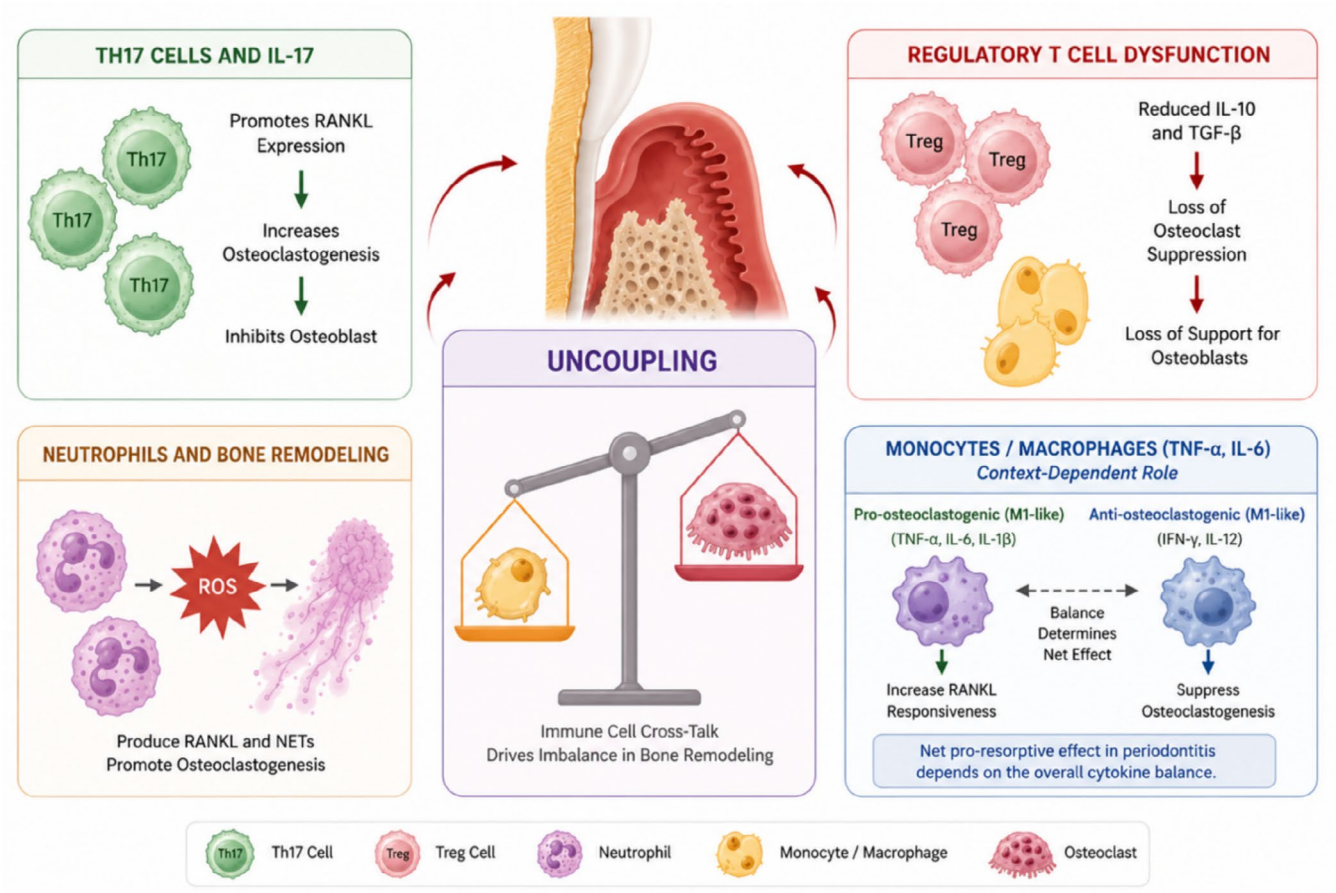
Immune‐driven mechanisms contributing to uncoupled bone remodeling in periodontitis. Schematic representation of the immune cellular interactions that disrupt the balance between bone resorption and bone formation in the periodontal microenvironment. Th17 cells produce IL‐17A, which promotes RANKL expression, enhances osteoclastogenesis, and inhibits osteoblast function. Neutrophils contribute to remodeling imbalance by releasing ROS, RANKL, and NETs, thereby amplifying inflammatory signaling. Treg dysfunction reduces the availability of anti‐inflammatory cytokines such as IL‐10 and TGF‐β, leading to diminished suppression of osteoclast activity and impaired support for osteoblasts. Inflammatory myeloid cells further promote osteoclastogenesis by producing pro‐inflammatory cytokines and increasing RANKL responsiveness. Together, these interconnected immune pathways drive remodeling uncoupling, favoring bone resorption over bone formation and contributing to progressive alveolar bone loss during periodontitis. NETs, neutrophil extracellular traps; RANKL, receptor activator of nuclear factor kappa B ligand; ROS, reactive oxygen species. Created with BioRender.com (license no. GZ29ECUCVK).

Bone remodeling in inflammatory diseases is increasingly understood through the context of osteoimmunology, which recognizes that immune cells are not merely bystanders in bone loss but active regulators of skeletal homeostasis. In periodontitis, chronic immune activation profoundly reshapes the cellular and molecular environment of alveolar bone, promoting remodeling uncoupling [17, 29]. This imbalance arises from coordinated dysfunction across multiple immune cell populations, collectively driving remodeling uncoupling (Figure 3).

### 7.2 Th17 Cells and IL‐17A‐Driven Uncoupling

Among adaptive immune cells, Th17 cells play a central role in periodontal bone loss. Th17 cells accumulate in periodontal lesions and produce high levels of IL‐17A, a cytokine with potent effects on both immune and skeletal cells [26, 108, 164]. IL‐17A reinforces the inflammatory microenvironment and contributes to remodeling imbalance by simultaneously promoting osteoclast activity and impairing osteoblast function [26, 108, 164]. Experimental studies have shown that IL‐17A suppresses osteoblast differentiation and mineralization while enhancing inflammatory signaling within osteogenic cells [165, 166]. Consequently, IL‐17A‐dominated immune responses create a microenvironment in which resorption proceeds without effective anabolic compensation. This dual effect distinguishes IL‐17A from cytokines that primarily stimulate osteoclasts and underscores its role as a central driver of uncoupled remodeling in periodontitis.

### 7.3 Treg Dysfunction

In healthy tissues, regulatory T cells (Tregs) provide an essential counterbalance to pro‐inflammatory immune responses and contribute to skeletal homeostasis by limiting osteoclast differentiation and supporting bone formation. Tregs suppress osteoclastogenesis through both cell‐contact‐dependent mechanisms and the secretion of anti‐inflammatory cytokines, including IL‐10 and TGF‐β [167]. These cytokines dampen the activity of pro‐inflammatory immune cells and reduce the overall inflammatory tone within the tissue. IL‐10 limits the activation of macrophages and effector T cells, thereby decreasing the production of pro‐resorptive signals. TGF‐β contributes to immune regulation while also supporting tissue repair processes, including the maintenance of mesenchymal cell function [168, 169, 170]. Through these combined actions, Tregs create a microenvironment that restrains excessive bone resorption and allows normal remodeling to proceed.

At the molecular level, Treg identity and function are controlled by the transcription factor FoxP3, which governs their suppressive phenotype and stability. Sustained expression of FoxP3 is essential for maintaining Treg function over time. In stable conditions, FoxP3‐driven activation ensures that Tregs remain effective regulators of immune responses, continuously counterbalancing pro‐inflammatory signals [171, 172]. However, this regulatory function is sensitive to the surrounding inflammatory environment. In periodontitis, Treg function appears to be both quantitatively and qualitatively impaired. Although Tregs may be present within periodontal lesions, their suppressive capacity is reduced in the setting of persistent inflammation, allowing Th17‐dominated responses to prevail [108, 172, 173, 174]. Inflammatory cytokines can reduce FoxP3 expression or alter Treg behavior, weakening their ability to control immune activation. In some cases, Tregs may even lose their regulatory phenotype and adopt features of pro‐inflammatory cells, further contributing to immune imbalance. This imbalance not only enhances osteoclastogenic signaling but also removes an important restraint on inflammation‐induced inhibition of osteoblast activity [108, 172, 173, 174]. The resulting loss of immune‐mediated control contributes to sustained remodeling uncoupling.

### 7.4 Neutrophils as Modulators of Bone Remodeling

Neutrophils are the most abundant immune cells in periodontal tissues and represent a defining feature of periodontitis. Although traditionally viewed as short‐lived antimicrobial effector cells, neutrophils are now recognized as important modulators of bone remodeling under inflammatory conditions [175]. Activated neutrophils can express RANKL, thereby directly contributing to osteoclast differentiation in inflamed tissues [176, 177]. In periodontitis, where neutrophil infiltration is persistent, this additional source of RANKL further amplifies osteoclastogenic signaling.

Beyond their role in promoting bone resorption, neutrophils also interfere with effective bone formation. Activated neutrophils release reactive oxygen species and a range of proteolytic enzymes as part of their antimicrobial function. When produced in excess, these molecules damage the extracellular matrix and alter the local tissue environment [178, 179, 180, 181]. This not only compromises structural integrity but also affects the survival and function of osteoblast‐lineage cells. Under these conditions, osteoblasts are exposed to a hostile environment that limits their ability to differentiate, produce matrix, and contribute to tissue repair [178, 179, 180, 181].

A particularly relevant mechanism is the formation of neutrophil extracellular traps (NETs)—networks of DNA and associated proteins released in response to strong inflammatory stimuli. While NETs contribute to microbial containment, they also promote local oxidative stress and tissue injury, and NET‐associated molecules can impair osteoblast differentiation while enhancing osteoclast activation, thereby reinforcing resorptive dominance while limiting skeletal repair [182, 183, 184]. In the periodontal environment, where inflammatory stimuli are continuous, NET formation is often prolonged and poorly regulated, sustaining tissue‐stress signals that bridge immune activation and skeletal imbalance, simultaneously supporting resorptive processes and limiting repair [175, 185].

### 7.5 Myeloid Cell Skewing Toward Osteoclastogenic Phenotypes

Chronic inflammation also alters the differentiation trajectory of myeloid progenitor cells, biasing them toward osteoclastogenic phenotypes. Under normal conditions, circulating monocytes can differentiate into a range of cell types, including macrophages that support tissue defense and repair. This differentiation process is tightly regulated and adapts to the needs of the tissue [22, 29, 38, 186]. In periodontitis this balance is disrupted. Persistent exposure to inflammatory signals and microbial products reshapes the local environment in a way that favors differentiation toward osteoclast‐forming cells. Monocytes and macrophages in periodontal lesions are continuously exposed to inflammatory cytokines and microbial products that enhance their responsiveness to RANKL and favor osteoclast differentiation over alternative macrophage fates [22, 29, 38, 186]. This skewing of the myeloid compartment expands the pool of osteoclast precursors and sustains osteoclast formation even when resorptive demand would normally decline. Importantly, inflammatory macrophages also produce cytokines that inhibit osteoblast differentiation and survival, thereby linking myeloid activation to both arms of remodeling uncoupling. Immune‐driven changes in myeloid cell fate therefore contribute not only to excessive bone resorption but also to persistent suppression of bone formation.

## 8 Periodontitis as a Uniquely Accessible Model of Inflammatory Uncoupling In Vivo

A central challenge in skeletal biology is to investigate inflammatory uncoupling of bone remodeling under conditions that preserve physiological context while allowing longitudinal analysis. Many disorders characterized by remodeling imbalance, including RA, osteoporosis, impaired fracture repair, and skeletal aging, affect anatomical sites that are difficult to repeatedly access in humans, making mechanistic inference often depend on indirect biomarkers or end‐stage surgical specimens.

In this regard, periodontitis offers a distinctive advantage, as it occurs within a spatially confined skeletal compartment that is clinically accessible and amenable to repeated, minimally invasive sampling. Gingival crevicular fluid (GCF), in particular, can be readily collected chairside and reflects local host‐microbial interactions and inflammatory activity at the tooth‐gingiva‐bone interface, thereby supporting longitudinal assessment of molecular mediators directly implicated in remodeling uncoupling and treatment response [187, 188, 189]. This accessibility enables longitudinal human in vivo studies linking local inflammation to remodeling‐related pathways, including cytokine networks and regulators of osteoclastogenesis and osteoblast suppression [55, 187, 188, 189].

Beyond access to GCF, periodontitis provides a localized and anatomically well‐defined inflammatory bone lesion in which the relationships among immune activation, bone‐resident cell responses, and structural outcomes can be examined at high spatial resolution. In contrast to systemic skeletal diseases, in which bone loss is diffuse and influenced by endocrine, metabolic, and age‐related factors, periodontal bone loss allows evaluation of how persistent local inflammatory signaling perturbs coupling within a discrete region of bone undergoing continuous turnover and mechanoadaptation [22, 54, 64]. Importantly, accumulating evidence supports a key role for investigating how osteocytes integrate inflammatory and mechanical signals and how this integration reshapes the balance between RANKL and Wnt signaling [54, 55, 58, 59, 64, 144].

The value of periodontitis as an in vivo model is further enhanced by its chronicity. Whereas acute injuries, including uncomplicated fractures, typically involve a transient inflammatory phase followed by restoration of coordinated remodeling, periodontitis imposes persistent immune activation sustained by dysbiotic biofilms, creating a stable inflammatory microenvironment in which coupling failure can become sustained and self‐reinforcing across time and sites [20, 22, 38], even if the rate and intensity of active bone loss vary among individuals and locations as reflected in the disease grading classification. Chronic inflammation and oxidative stress are widely recognized to impair osteoblast differentiation, reduce osteogenesis, and promote osteocyte apoptosis, mechanisms that contribute to net bone loss by enhancing resorption while suppressing formation [144, 190]. This chronic inflammatory setting makes the periodontium particularly informative for understanding how prolonged cytokine exposure and redox imbalance progressively extinguish anabolic competence and disrupt the resorption‐to‐formation transition within BMUs.

Mechanistic insights derived from periodontitis also extend beyond oral biology with clear relevance to osteoporosis, where coupling failure at the reversal‐to‐formation transition and Wnt‐antagonist–driven suppression of bone formation parallel the mechanisms seen in periodontal lesions [191, 192, 193, 194]. Because periodontitis features strong osteocyte involvement in both RANKL induction and Wnt antagonism, it provides an accessible setting to examine how inflammatory signals reshape osteocyte outputs that govern coupling efficiency [54, 55, 64, 123, 195]. In contrast to ephrin and semaphorin signaling, Wnt antagonism has more direct periodontal support [123, 128, 130, 150, 158, 160, 180, 196], with increased sclerostin and DKK1 reported in periodontal tissues, GCF, serum, and experimental periodontitis models.

Periodontitis also shares mechanistic parallels with failed fracture repair, in which successful healing depends on a coordinated inflammatory response followed by a timely transition to osteogenesis and remodeling [197, 198]. Because periodontitis is highly prevalent and often more severe with aging, it further provides an accessible setting in which to examine how inflammaging‐related mechanisms converge on remodeling uncoupling in vivo, as discussed in Section 9.3 [36, 144, 195, 199, 200].

Taken together, these features, localized and repeatedly accessible inflammatory osteolysis, substantial osteocyte involvement, and sustained immune activation, establish periodontitis as one of the most biologically informative in vivo models of inflammatory remodeling uncoupling, with relevance extending to other systemic bone conditions, and may help guide therapeutic strategies aimed at restoring physiological coupling.

## 9 Integration of Osteocyte Biology, Skeletal Stem Cells, Mechanobiology, and Aging

**FIGURE 4:**
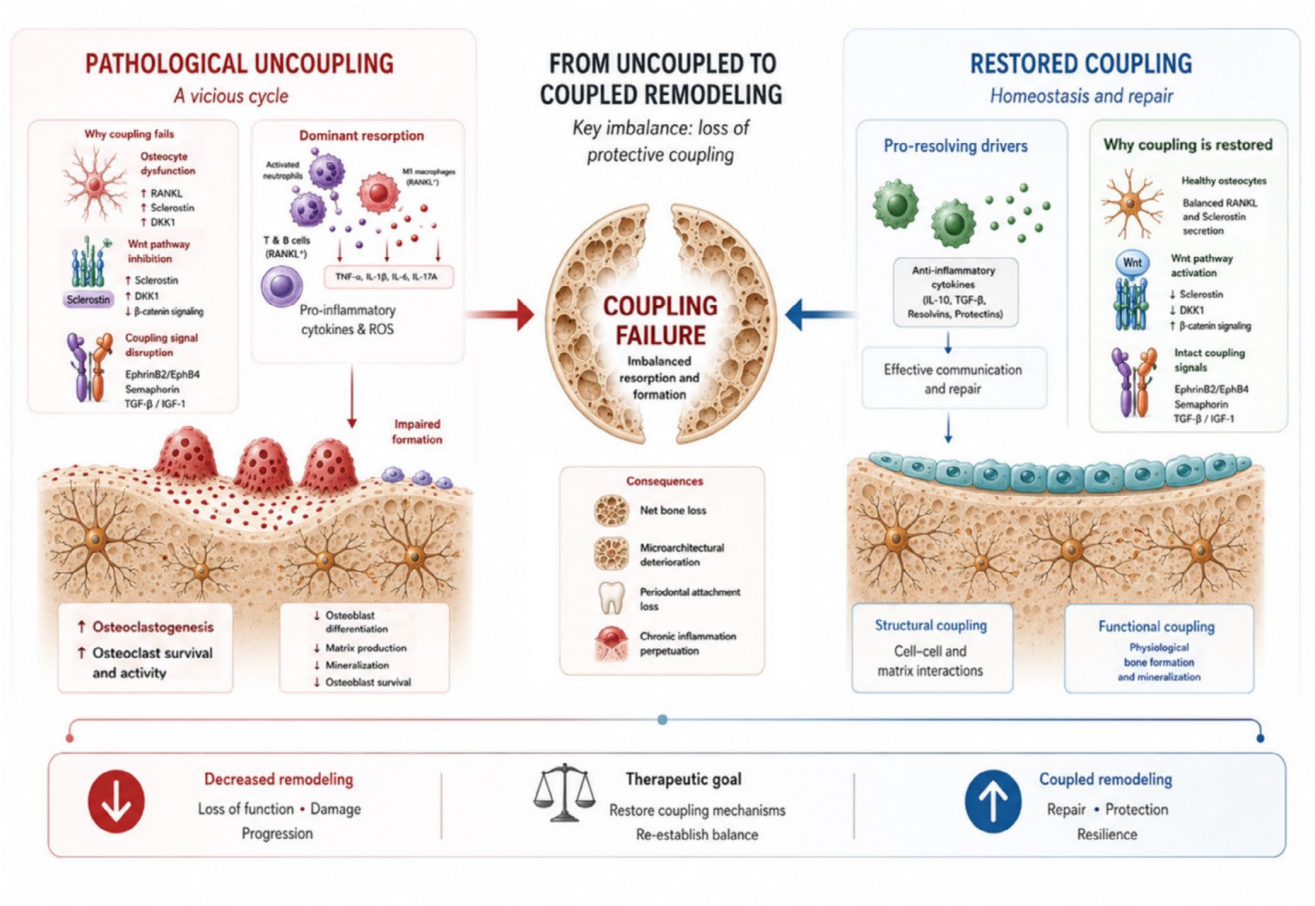
Unified model of inflammatory uncoupling of bone remodeling. Conceptual model of pathological uncoupling and restoration of coupled bone remodeling in inflammatory bone loss. The left panel illustrates the mechanisms driving pathological uncoupling in periodontitis and related inflammatory skeletal diseases. Persistent inflammatory activation promotes a resorption‐dominant microenvironment characterized by osteocyte dysfunction, increased RANKL expression, NF‐κB and MAPK activation, Wnt/β‐catenin inhibition, and disruption of key osteoclast–osteoblast coupling signals, including ephrinB2/EphB4 and semaphorin pathways. Multiple immune cell populations, including neutrophils, macrophages, T cells, and B cells, contribute to sustained inflammatory cytokine production and osteoclast hyperactivation. Concomitantly, osteoblast differentiation, matrix production, mineralization, and survival are suppressed, resulting in ineffective bone formation and progressive bone loss. The right panel illustrates restoration of remodeling balance through resolution of inflammation and preservation of physiological coupling mechanisms. Reduced inflammatory signaling, normalization of the RANKL/OPG balance, restoration of Wnt/β‐catenin activity, preservation of osteocyte function, and recovery of osteoclast–osteoblast communication collectively re‐establish coordinated bone resorption and formation, thereby supporting maintenance of skeletal integrity and tissue regeneration. DKK1, Dickkopf‐1; IGF‐1, insulin‐like growth factor‐1; MAPK, mitogen‐activated protein kinase; NF‐κB, nuclear factor kappa B; OPG, osteoprotegerin; RANKL, receptor activator of nuclear factor kappa B ligand; TGF‐β, transforming growth factor‐beta. Created with BioRender.com (license no. GZ29ECUCVK).

A comprehensive understanding of inflammatory uncoupling of bone remodeling requires integration of regulatory systems beyond osteoclasts and osteoblasts alone (Figure 4). While several of the mechanisms discussed throughout this review are directly supported by experimental and clinical studies in periodontitis [22, 54, 55, 64, 123, 150, 160], others derive from advances in osteocyte biology, skeletal stem cell research, mechanobiology, and inflammatory skeletal diseases outside the periodontal field [43, 45, 46, 50, 145, 191, 192, 201]. These concepts provide a useful mechanistic context for understanding periodontal bone loss but, in some cases, should be regarded as biologically plausible hypotheses that warrant direct validation in periodontal tissues (Table 1).

**TABLE 1:** Mechanisms proposed to contribute to inflammatory uncoupling of bone remodeling in periodontitis, classified by the strength and origin of supporting periodontal evidence.

Mechanism/pathway	Role in uncoupling	Direct periodontal evidence	Strength of periodontal support	Representative references
RANKL dominance and excessive osteoclastogenesis	Resorption	Yes—periodontal tissue, GCF, experimental models	Strong	[1, 17, 29, 54, 55, 95]
Pro‐resorptive cytokines (TNF‐α, IL‐1β, IL‐6, IL‐17A)	Resorption	Yes—direct periodontal	Strong	[17, 106, 107, 108, 109, 110, 164]
NF‐κB/MAPK activation	Resorption	Yes—periodontal + skeletal	Strong	[17, 29, 111, 119]
Suppression of Runx2/Osterix, osteoblast inhibition	Formation	Periodontal experimental	Moderate	[119, 120, 121, 122]
Wnt/β‐catenin antagonism (sclerostin, DKK1)	Formation	Yes—↑SOST/DKK1 in tissue, GCF, serum, models	Moderate	[123, 128, 130, 150, 158]
Sclerostin paradox (TNF‐α → SOST in surviving osteocytes)	Formation	Single line (infliximab, ligature + diabetes rat) → hypothesis	Limited	[159, 160]
Osteocyte apoptosis as proximal trigger	Coupling/initiation	Emerging periodontal	Moderate–limited	[54, 55, 130, 190]
Panx1/ATP/P2X7 bystander signaling	Coupling/initiation	Microdamage and long‐bone models	Limited–partly extrapolated	[52, 53]
ephrinB2/EphB4 coupling	Resorption to formation transition	Extrapolated; periodontal data limited	Limited–partly extrapolated	[145, 146]
Semaphorin 3A/4D signaling	Coupling	Extrapolated; periodontal data limited	Limited–partly extrapolated	[67, 68]
Skeletal stem/progenitor dysfunction	Formation	Broader skeletal biology	Limited–partly extrapolated	[201, 202, 203, 204, 205]
Mechanotransduction disruption	Adaptive remodeling	Osteocyte mechanobiology established broadly; periodontal emerging	Moderate–partly extrapolated	[87, 114, 206, 207, 208]
Aging/inflammaging amplification	Modifier	Broader skeletal	Partly extrapolated	[36, 195, 199, 200, 201]
Host–microbe/barrier‐driven amplification	Modifier (periodontitis‐unique)	Direct periodontal	Strong (periodontitis‐specific)	[20, 21, 37, 38, 39]

*Note:* Strength of periodontal support is defined as follows: strong—supported by direct periodontal evidence from human clinical or observational studies and/or experimental periodontitis models; moderate—supported by periodontal experimental data but with limited clinical validation; limited—few direct periodontal studies available, with the mechanism presented as a biologically plausible hypothesis requiring periodontal‐specific testing; partly extrapolated—evidence derived predominantly from broader skeletal biology, other skeletal diseases (e.g., rheumatoid arthritis, osteoporosis, fracture repair), or non‐periodontal anatomical sites.

Abbreviations: ATP, adenosine triphosphate; DKK1, Dickkopf‐1; EphB4, ephrin type‐B receptor 4; IL, interleukin; MAPK, mitogen‐activated protein kinase; NF‐κB, nuclear factor kappa B; NFATc1, nuclear factor of activated T cells c1; P2X7, P2X purinoceptor 7; Panx1, pannexin‐1; RANKL, receptor activator of nuclear factor kappa B ligand; Runx2, runt‐related transcription factor 2; Sema, semaphorin; SOST, sclerostin (gene); TNF, tumor necrosis factor; Wnt, wingless‐related integration site.

### 9.1 Skeletal Stem and Progenitor Cells as Determinants of Regenerative Capacity

Effective remodeling coupling depends on the recruitment and differentiation of skeletal stem and progenitor cells capable of generating new osteoblasts. These progenitors reside within bone marrow, periosteum, and perivascular niches and constitute the principal source of osteoblast‐lineage cells required for bone regeneration [148, 202, 209]. Osteoclast‐mediated resorption releases growth factors such as TGF‐β and IGF‐1, which recruit skeletal progenitors and promote osteogenic differentiation, thereby restoring bone mass and structural integrity [51, 148].

Inflammatory signaling disrupts skeletal progenitor cell function at multiple levels. It alters skeletal progenitor cell function by limiting osteogenic differentiation capacity and promoting alternative cell fates, thereby reducing the availability of osteoblast‐lineage cells required for effective bone formation [138, 203, 210, 211]. In addition, inflammatory signaling promotes progenitor cell senescence and impairs regenerative capacity. Chronic inflammation and oxidative stress reduce skeletal stem cell self‐renewal and osteogenic potential, contributing to persistent failure of bone formation in inflammatory and aging‐related skeletal diseases [201, 204, 212]. These effects directly compromise the formation phase of remodeling and represent a key mechanism underlying sustained remodeling uncoupling. However, the contribution of skeletal stem and progenitor cell dysfunction to inflammatory uncoupling is strongly supported by skeletal regeneration and aging studies [138, 201, 203, 204, 210, 211, 212], but direct periodontal evidence remains comparatively limited.

### 9.2 Mechanobiology and Disruption of Adaptive Remodeling

Mechanical loading plays a central role in maintaining skeletal integrity by regulating osteocyte signaling and osteoblast activation [1, 18, 87]. Osteocytes detect mechanical strain and regulate bone formation through mechanotransduction pathways, including modulation of sclerostin expression and activation of Wnt/β‐catenin signaling [205, 206]. Mechanical loading suppresses sclerostin expression, thereby promoting osteoblast differentiation and bone formation, whereas unloading has the opposite effect, leading to bone loss [205, 206].

Inflammatory signaling disrupts these mechanotransduction pathways, impairing bone's ability to respond appropriately to mechanical stimuli. Cytokine‐mediated upregulation of sclerostin and other inhibitory mediators blunts osteoblast activation despite ongoing mechanical loading [127, 207]. Inflammatory damage to the osteocyte lacunar–canalicular network further impairs signal transmission and coordination between osteocytes and osteoblast‐lineage cells, thereby weakening the adaptive remodeling response [208, 213]. These disturbances effectively decouple mechanical loading from anabolic bone formation and contribute to progressive skeletal deterioration in inflammatory conditions. Importantly, mechanotransduction through osteocytes and Wnt/sclerostin signaling is well established in skeletal biology [87, 149, 151, 205, 206, 207], whereas direct evidence defining how periodontal inflammation disrupts these pathways in alveolar bone remains emerging [1, 18, 87, 114].

### 9.3 Aging and Systemic Skeletal Disease as Amplifiers of Uncoupling

Aging and systemic skeletal disease further modify the biological context in which remodeling occurs, thereby amplifying susceptibility to uncoupling [201]. Aging compromises remodeling efficiency through chronic inflammation, oxidative stress, reduced osteogenesis, impaired function, and altered osteocyte and skeletal stem cell function [36, 144, 195, 199, 200]. In parallel, systemic skeletal diseases such as osteoporosis are characterized not only by increased resorption but also by impaired reversal‐to‐formation transitions and deficient osteoblast‐mediated refilling of resorption cavities [36, 144, 195, 199, 200]. These systemic influences are highly relevant to inflammatory bone diseases because they reduce the bone capacity to recover once inflammation has perturbed coupling. Age‐related increases in osteocyte‐derived Wnt antagonists, reduced progenitor competence, and diminished anabolic responsiveness create a permissive background in which inflammatory cues exert disproportionately deleterious effects on remodeling balance [36, 144, 195, 199, 200]. Thus, aging and systemic skeletal disease should not be viewed merely as comorbidities, but as biological states that intensify and stabilize inflammatory remodeling failure.

### 9.4 The Unique Host–Microbe Context of Periodontal Remodeling Failure

As described in Section 3.3, periodontitis develops within a unique host–microbe ecosystem in which the periodontal tissues are continuously exposed to a diverse subgingival biofilm—an ongoing microbial trigger that distinguishes it from the relatively sterile environments of most other inflammatory skeletal disorders [21, 37, 39].

The gingival epithelium serves as a critical barrier separating the subgingival biofilm from underlying tissues. In health, epithelial cells contribute to tissue homeostasis through production of antimicrobial peptides, maintenance of junctional integrity, and regulation of immune surveillance [39, 40, 214]. During dysbiosis, however, microbial virulence factors, proteolytic enzymes, and inflammatory mediators compromise epithelial barrier function, increasing tissue permeability and facilitating deeper microbial penetration. This process amplifies local cytokine production, leukocyte recruitment, and osteoclastogenic signaling.

Importantly, microbial dysbiosis alone is insufficient to explain periodontal bone loss. Instead, disease progression reflects a maladaptive host response in which inflammatory pathways that normally protect against microbial challenge become chronically activated and ultimately disrupt tissue homeostasis [20, 21, 22, 38]. In this regard, periodontitis exemplifies a broader biological principle whereby persistent inflammatory stimuli interfere with the mechanisms that coordinate bone resorption and bone formation [22, 43]. However, the continuous presence of a microbial trigger represents an important distinction from other skeletal conditions [41, 43, 215, 216]. Therefore, periodontitis should be viewed not as a direct surrogate for all inflammatory bone diseases, but rather as a complementary and uniquely accessible model through which the interactions among microbial challenge, barrier dysfunction, immune regulation, and bone remodeling can be investigated in vivo.

### 9.5 Implications for Understanding Remodeling Failure

Disruptions across these interconnected domains—osteocyte signaling, skeletal stem cell function, mechanotransduction, and the aging, host–microbe, and systemic context—transform remodeling from a self‐limited repair process into a persistent pathological state [58, 148, 149, 217]. This integrated perspective underscores the need for therapeutic approaches that restore this physiological coupling.

## 10 Therapeutic Implications: Restoring Coupling Instead of Only Blocking Resorption

### 10.1 Moving Beyond Anti‐Resorptive Therapies

**FIGURE 5:**
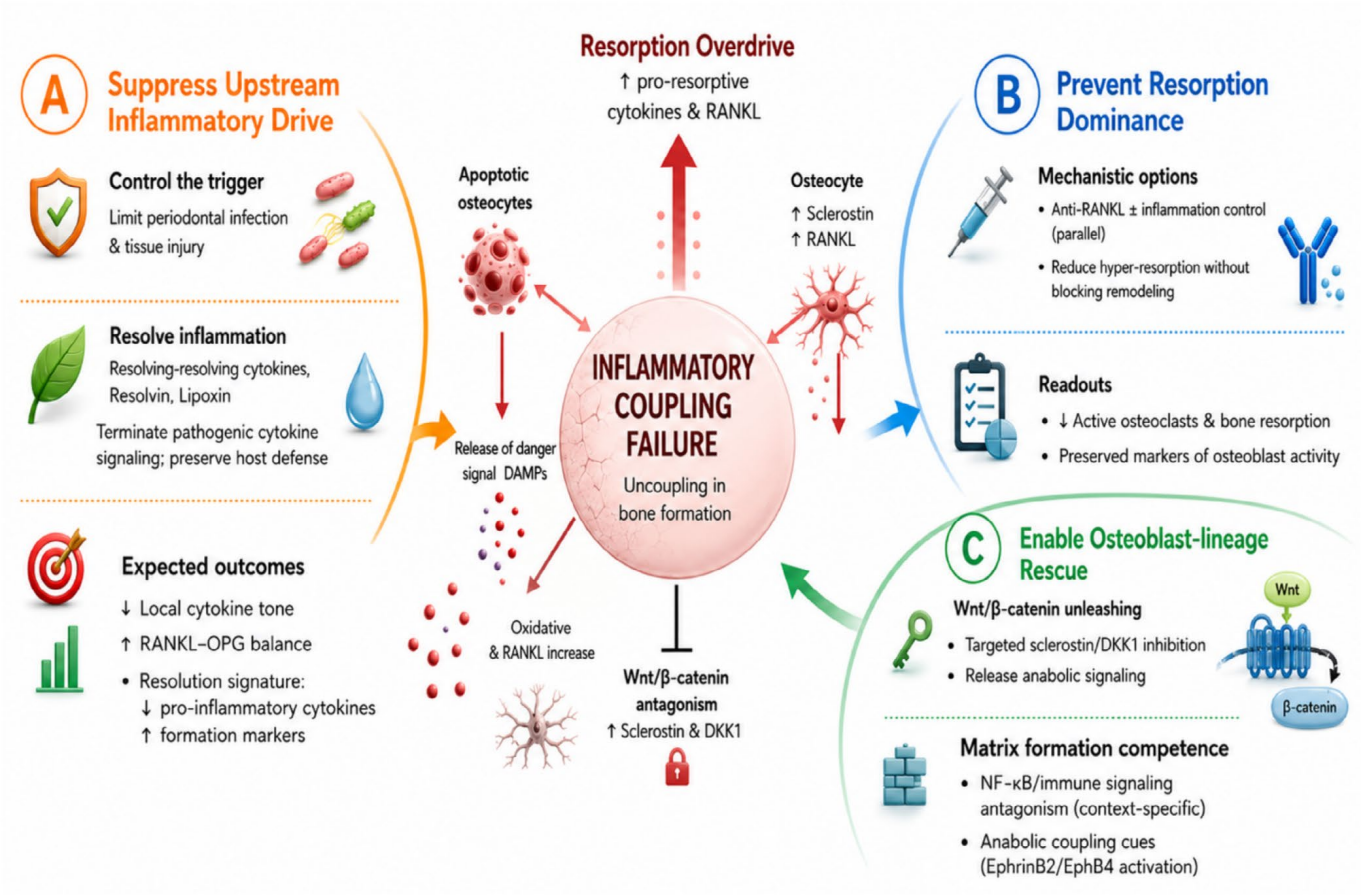
Unified model of inflammatory coupling failure and therapeutic restoration in periodontitis and inflammatory bone diseases. Schematic representation of inflammation‐driven uncoupling of bone remodeling and potential strategies to restore physiological coupling. Central panel: chronic inflammatory activation promotes a resorption‐dominant state characterized by increased pro‐resorptive cytokines and RANKL signaling, osteocyte dysfunction (↑ sclerostin and ↑ RANKL), osteocyte apoptosis, and Wnt/β‐catenin antagonism (sclerostin and DKK1), culminating in impaired osteoblast‐mediated bone formation (“coupling failure”). (A) Attenuate upstream inflammatory drive: periodontitis‐specific trigger control through biofilm reduction and mitigation of host tissue injury, combined with activation of resolution pathways by specialized pro‐resolving mediators such as resolvins and lipoxins, lowers pathogenic cytokine tone while preserving host defense. Expected readouts include restoration of the RANKL/OPG balance, reduction of pro‐inflammatory cytokines, and recovery of bone formation markers. (B) Prevent resorption dominance: mechanistically aligned anti‐RANKL strategies, combined with inflammation control, reduce osteoclast hyperactivation without fully suppressing physiological remodeling. Expected outcomes include reduced numbers of active osteoclasts and decreased bone resorption, with preservation of osteoblast activity. (C) Enable osteoblast‐lineage rescue: reactivation of anabolic Wnt/β‐catenin signaling by relieving pathway inhibition (e.g., through sclerostin or DKK1 blockade), together with context‐specific modulation of NF‐κB/immune signaling and restoration of coupling cues such as ephrinB2/EphB4, promotes osteogenic competence and matrix‐forming capacity. DKK1, Dickkopf‐1; OPG, osteoprotegerin; RANKL, receptor activator of nuclear factor kappa B ligand. Created with BioRender.com (license no. GZ29ECUCVK).

Current therapeutic strategies for inflammatory bone loss have largely focused on suppressing osteoclast activity. Although this approach can effectively reduce bone resorption, it does not address the fundamental impairment of bone formation that defines uncoupled remodeling in periodontitis. Insights from skeletal biology and osteoimmunology increasingly suggest that durable restoration of alveolar bone requires interventions that re‐establish the physiological dialog between bone resorption and bone formation (Figure 5).

The therapeutic strategies discussed in this section differ substantially in their stage of development and level of supporting evidence (Table 2). While conventional periodontal therapy and certain host‐modulation approaches have established clinical applicability, other interventions, including Wnt‐pathway modulation, sclerostin inhibition, SPMs, and strategies aimed at restoring physiological coupling mechanisms, remain largely supported by preclinical and translational research. The rationale for these approaches stems from advances in osteoimmunology and skeletal biology, which have demonstrated that targeting osteoclast activity, inflammatory pathways, Wnt signaling, and osteocyte‐derived regulatory networks can alter the balance between bone resorption and bone formation. Although periodontitis presents unique challenges related to persistent microbial stimulation and barrier disruption, these discoveries provide an important conceptual basis for the development of future therapies designed not only to suppress tissue destruction but also to restore remodeling homeostasis.

**TABLE 2:** Therapeutic strategies targeting inflammatory uncoupling of bone remodeling in periodontitis, organized by translational maturity and level of supporting evidence.

Strategy	Primary target(s)	Arm addressed	Best periodontal evidence	Representative findings	Translational status	References
Subantimicrobial‐dose doxycycline	MMPs, collagen degradation	Resorption	Human RCT	↓ collagenase and attachment loss; possible ↓ alveolar bone loss	Established	[218, 219, 220]
Anti‐resorptive (anti‐RANKL, bisphosphonates)	Osteoclast differentiation/activity	Resorption	Established systemically; periodontal animal	Suppress resorption; little direct anabolic effect	Established (systemic)/preclinical (perio)	[221, 222, 223, 224, 225] (systemic efficacy) [226, 227, 228] (periodontal animal); MRONJ caveat [229, 230, 231, 232, 233]
Cytokine inhibition (anti‐TNF, anti‐IL‐1)	TNF‐α, IL‐1β	Resorption	Experimental periodontitis	↓ infiltrate and osteoclastic loss; limited perio clinical	Preclinical	[234, 235] (+27 for combined strategies)
Wnt‐pathway modulation	DKK1, Wnt5a/sFRP5, β‐catenin	Formation	Animal + observational	↓ bone loss; restored anabolic signaling	Preclinical/translational	[123, 236, 237, 238, 239] (periodontal); broader rationale [240, 241, 242, 243, 244]
Sclerostin inhibition	SOST, Wnt/β‐catenin	Formation	Animal + observational	↑ regeneration and bone volume	Preclinical	[237, 245, 246, 247, 248, 249, 250, 251, 252, 253, 254, 255, 256, 257, 258]
Specialized pro‐resolving mediators	Resolution pathways, neutrophil/macrophage	Resolution + coupling	Animal + translational	↓ inflammation/dysbiosis/bone loss; partial regeneration	Preclinical/translational	[27, 28, 133, 135, 136, 137, 218, 259, 260, 261, 262, 263, 264]
Immunomodulation (Treg/Th17, IL‐10, TGF‐β)	Immune‐cell balance	Resorption + coupling	Animal/mechanistic	↓ osteoclastogenesis; promotes repair	Investigational	[21, 27, 108, 134, 172, 173, 174, 235]
Intracellular signaling modulation	Inflammatory signaling	Resorption	Preclinical	Attenuates inflammation and osteoclast activation	Investigational/conceptual	[27, 108, 235]

*Note:* Levels of periodontal evidence are defined as: human RCT—randomized controlled trial in periodontal patients; human observational—clinical or biomarker association studies; experimental periodontitis—in vivo animal models of periodontal disease; in vitro/mechanistic—cell‐culture or molecular studies; extrapolated—evidence derived primarily from non‐periodontal skeletal tissues or diseases. Translational status is categorized as: established—in current clinical use or clinically validated; preclinical—supported predominantly by animal and/or in vitro data without periodontal clinical trials; investigational/conceptual—mechanistically supported or emerging strategies not yet evaluated as periodontal therapies.

Abbreviations: DKK1, Dickkopf‐1; GCF, gingival crevicular fluid; IL, interleukin; MMP, matrix metalloproteinase; OPG, osteoprotegerin; RANKL, receptor activator of nuclear factor kappa B ligand; RCT, randomized controlled trial; SDD, subantimicrobial‐dose doxycycline; sFRP5, secreted frizzled‐related protein 5; SOST, sclerostin (gene); SPM, specialized pro‐resolving mediator; TGF‐β, transforming growth factor beta; Th17, T helper 17 cell; TNF, tumor necrosis factor; Treg, regulatory T cell; Wnt, wingless‐related integration site.

### 10.2 Limitations of Anti‐Resorptive Strategies

Bisphosphonates and other anti‐resorptive agents have demonstrated clear efficacy in reducing bone turnover and fracture risk in systemic skeletal diseases [221, 222, 223, 224, 265]. Their primary mechanism, inducing osteoclast apoptosis and suppressing bone resorption, effectively slows bone loss and stabilizes skeletal structure. However, when these strategies are applied to oral inflammatory conditions such as periodontitis, important biological and clinical limitations become evident. By inducing osteoclast apoptosis and suppressing bone resorption, bisphosphonates can effectively limit further bone loss; however, they do not promote new bone formation or repair of pre‐existing bone defects [225].

A major concern specific to the craniofacial skeleton is the risk of medication‐related osteonecrosis of the jaw (MRONJ). The jawbones are characterized by high turnover, constant mechanical loading, and frequent exposure to microbial challenge. Suppression of remodeling in this context compromises bone's ability to adapt to injury and infection, thereby predisposing to necrosis and impaired healing [229, 230, 231, 232]. This risk underscores a fundamental limitation of prolonged anti‐resorptive therapy in oral tissues. Even when MRONJ does not occur, anti‐resorptive strategies often yield incomplete regeneration. Suppressing osteoclast activity may reduce additional bone loss, but it does not activate osteoblast‐mediated repair [229, 230, 231]. In inflammatory environments in which osteoblast differentiation and function are already compromised, this imbalance may stabilize existing defects instead of restoring normal bone architecture. These limitations collectively reframe antiresorptive therapy in the oral context not merely as a strategy with manageable risks, but as a paradigm with fundamental incompatibility with the biology of alveolar bone.

Importantly, current standard periodontal therapy consisting of mechanical debridement, biofilm disruption, and targeted anti‐inflammatory or antimicrobial adjuncts does not act primarily on osteoclasts but on the upstream infectious and inflammatory stimulus [209]. By reducing microbial dysbiosis and attenuating the cytokine milieu that drives RANKL expression, current treatment indirectly regulates the RANKL/OPG balance and creates conditions permissive for coupled bone formation, without freezing remodeling or impairing the bone's adaptive capacity. This mechanistic alignment of current clinical practice with upstream inflammatory control provides indirect validation for the therapeutic agenda developed in Section 10.3 and underscores why the field's evolution away from antiresorptives toward pro‐resolution and coupling‐restoration strategies represents a scientifically coherent progression.

Importantly, anti‐resorptive therapies remain highly effective and evidence‐based interventions for osteoporosis and other metabolic bone diseases [221, 222, 223, 224, 265]. Their limitations in periodontitis do not diminish their clinical value in appropriate skeletal contexts but rather highlight the unique biological requirements of alveolar bone [226, 227, 233], where preservation of remodeling dynamics is particularly important.

### 10.3 Anabolic and Coupling‐Focused Strategies

#### 10.3.1 Wnt Signaling Modulation

Strategies that restore anabolic signaling pathways represent a promising approach to re‐establishing bone formation in inflammatory environments [228, 240, 241]. Inflammatory skeletal diseases are consistently associated with increased expression of Wnt antagonists, including sclerostin and DKK1, which suppress osteoblast activity [46, 129]. Modulation of this pathway, therefore, has the potential to re‐enable osteogenic responses even in tissues previously exposed to excessive bone resorption. Previous studies indicate that enhancing Wnt signaling can stimulate bone formation and partially restore remodeling coupling in inflammatory settings [242, 243, 244]. However, the pleiotropic nature of Wnt signaling requires careful regulation, as excessive or uncontrolled activation can lead to aberrant bone formation or off‐target effects [242, 243, 244].

Evidence from both human and experimental studies supports the concept that dysregulation of Wnt signaling contributes to the uncoupling of bone remodeling in periodontitis and may represent an interesting therapeutic target. In a clinical study, Napimoga et al. [123] demonstrated significantly increased gingival expression of the canonical Wnt antagonists sclerostin (SOST) and DKK1 in chronic periodontitis, with expression levels correlating positively with clinical disease severity. Elevated circulating sclerostin concentrations were also observed, suggesting that periodontal inflammation may suppress Wnt/β‐catenin‐mediated osteogenesis both locally and systemically. These findings support the concept that inflammatory bone loss is associated not only with enhanced osteoclastogenesis but also with active inhibition of anabolic signaling pathways required for bone formation.

In addition to canonical Wnt signaling, non‐canonical pathways also appear to contribute to periodontal destruction. Maekawa et al. [266] demonstrated that periodontitis lesions exhibit increased expression of Wnt5a and reduced expression of its endogenous antagonist, secreted frizzled‐related protein‐5 (sFRP5), resulting in a pro‐inflammatory signaling environment. In vitro, *Porphyromonas gingivalis* lipopolysaccharide upregulated Wnt5a while suppressing sFRP5 expression in gingival epithelial cells, leading to enhanced inflammatory cytokine production. Importantly, local administration of sFRP5 in ligature‐induced periodontitis significantly reduced inflammatory cytokine expression, osteoclast numbers, and alveolar bone loss. These findings suggest that modulation of the Wnt5a/sFRP5 axis may attenuate periodontal inflammation while simultaneously limiting osteoclastogenesis, highlighting an additional mechanism through which Wnt‐targeted therapies may restore balanced bone remodeling.

More recently, a systematic review and meta‐analysis of eight animal studies evaluating pharmacological or genetic inhibition of sclerostin provided preclinical evidence supporting Wnt‐targeted regenerative therapy in periodontitis [244]. Across diverse experimental models, sclerostin inhibition consistently reduced alveolar bone loss while increasing bone volume fraction, bone mineral density, and osteogenic markers such as osteocalcin. Mechanistically, these benefits were attributed to restoration of canonical Wnt/β‐catenin signaling, suppression of the RANKL/OPG imbalance, and disruption of inflammatory TNF‐α/NF‐κB/SOST feedback loops that promote osteocyte‐mediated bone resorption. Importantly, the meta‐analysis concluded that sclerostin inhibition not only preserved existing alveolar bone but also actively promoted periodontal bone regeneration, supporting the concept that restoring anabolic signaling may represent a more physiologically relevant strategy than approaches focused exclusively on suppressing resorption.

Collectively, these studies indicate that Wnt dysregulation in periodontitis extends beyond impaired osteoblast differentiation and involves complex interactions among osteocytes, inflammatory pathways, osteoclastogenesis, and tissue regeneration. Therapeutic modulation of Wnt signaling through inhibition of sclerostin or DKK1, enhancement of β‐catenin signaling, or regulation of non‐canonical Wnt5a/sFRP5 pathways may therefore represent encouraging host‐directed strategies aimed at restoring physiological coupling between bone resorption and formation. Although experimental periodontitis models support targeting Wnt antagonists [123, 236, 237, 238, 266], the long‐term safety of enhancing Wnt signaling at a chronically inflamed site remains uncertain, and its therapeutic application will require precise contextual control.

#### 10.3.2 Sclerostin Inhibition

Among the anabolic pathways disrupted during periodontitis, increasing attention has focused on the Wnt/β‐catenin signaling cascade and its endogenous inhibitor sclerostin. Produced primarily by osteocytes, sclerostin suppresses osteoblast differentiation and activity through inhibition of Wnt signaling, while also promoting a pro‐resorptive environment by increasing the RANKL/OPG ratio. Consequently, sclerostin inhibition has emerged as one of the most promising anabolic strategies in skeletal medicine [239, 245, 246, 247, 248, 249, 250, 251]. Neutralization of sclerostin enhances Wnt signaling, increases osteoblast activity, and may reduce osteocyte‐derived pro‐resorptive signals, thereby attending to both arms of remodeling uncoupling [46, 129, 252, 253].

Evidence supporting this concept first emerged from experimental studies demonstrating increased osteocytic sclerostin expression in periodontal lesions. Kim et al. [250] reported that ligature‐induced periodontitis increased the number of sclerostin‐positive osteocytes, while the combination of diabetes and periodontitis resulted in sustained sclerostin expression, persistent suppression of osteoid formation, and greater alveolar bone loss. Notably, these effects occurred despite similar osteoclast numbers, suggesting that impaired bone formation contributed substantially to disease progression. The authors further demonstrated a close association between elevated TNF‐α expression and increased osteocytic sclerostin levels, providing mechanistic evidence that inflammatory cytokines may suppress bone formation through osteocyte‐mediated pathways.

Clinical observations have supported these experimental findings. Chatzopoulos et al. [236, 254] demonstrated increased gingival crevicular fluid levels of sclerostin in patients with generalized moderate‐to‐severe chronic periodontitis, suggesting that local Wnt inhibition is associated with periodontal disease severity. Furthermore, Ozden et al. [255] reported significant alterations in gingival crevicular fluid levels of both sclerostin and DKK1 in postmenopausal women with osteoporosis and periodontitis, further implicating Wnt antagonists in periodontal bone destruction and suggesting potential interactions between local and systemic skeletal remodeling.

The strongest experimental evidence supporting sclerostin as a therapeutic target comes from studies evaluating pharmacological or genetic suppression of SOST signaling. In a landmark study, Taut et al. [256] demonstrated that systemic administration of a sclerostin‐neutralizing antibody (Scl‐Ab) after ligature‐induced periodontitis promoted substantial alveolar bone regeneration. Scl‐Ab treatment restored bone volume fraction and tissue mineral density to levels comparable to healthy controls while increasing systemic markers of bone formation, including osteocalcin and procollagen type I N‐terminal propeptide. Importantly, these regenerative effects occurred after disease establishment, indicating that anabolic stimulation can actively restore lost periodontal bone and not merely prevent further destruction.

Recent study made by Phanrungsuwan et al. [257] expanded these observations. Genetic deletion of Sost increases alveolar bone volume and enhances post‐extraction alveolar bone healing. Interestingly, while alveolar bone responded robustly to SOST deficiency, cellular cementum exhibited a comparatively limited response, suggesting tissue‐specific regulation of Wnt signaling within the periodontium. These findings further reinforce the concept that osteocytes are principal mediators of the anabolic response induced by sclerostin inhibition.

The translational significance of these findings has been highlighted by Liao et al. [251] who comprehensively reviewed the role of sclerostin throughout oral tissues. Beyond its effects on alveolar bone, sclerostin is expressed in periodontal ligament cells, cementocytes, odontoblasts, and dental pulp stem cells, suggesting broader regulatory functions in periodontal homeostasis and regeneration. The authors proposed that sclerostin inhibition may simultaneously promote osteogenesis, cementogenesis, dentin repair, and periodontal regeneration, making it a particularly attractive host‐modulation strategy for oral diseases characterized by impaired tissue repair.

Collectively, these studies indicate that the benefits of sclerostin inhibition extend beyond suppression of bone destruction and involve active restoration of anabolic remodeling through reactivation of Wnt/β‐catenin signaling, normalization of the RANKL/OPG balance, and interruption of inflammatory feedback loops. These findings support the emerging concept that restoring bone formation may be more biologically appropriate than exclusively suppressing osteoclast activity in periodontitis. Consequently, therapies targeting sclerostin may represent one interesting anabolic approach for re‐establishing physiologic coupling between bone resorption and bone formation in periodontitis.

However, no periodontal‐specific clinical trials of anti‐sclerostin therapy are yet available, and also because sclerostin regulates bone turnover and mechanoadaptation, its inhibition at a chronically inflamed, microbially challenged site may yield context‐dependent outcomes that warrant a cautious, site‐specific approach.

#### 10.3.3 Pro‐Resolving Mediators (SPM)

**FIGURE 6:**
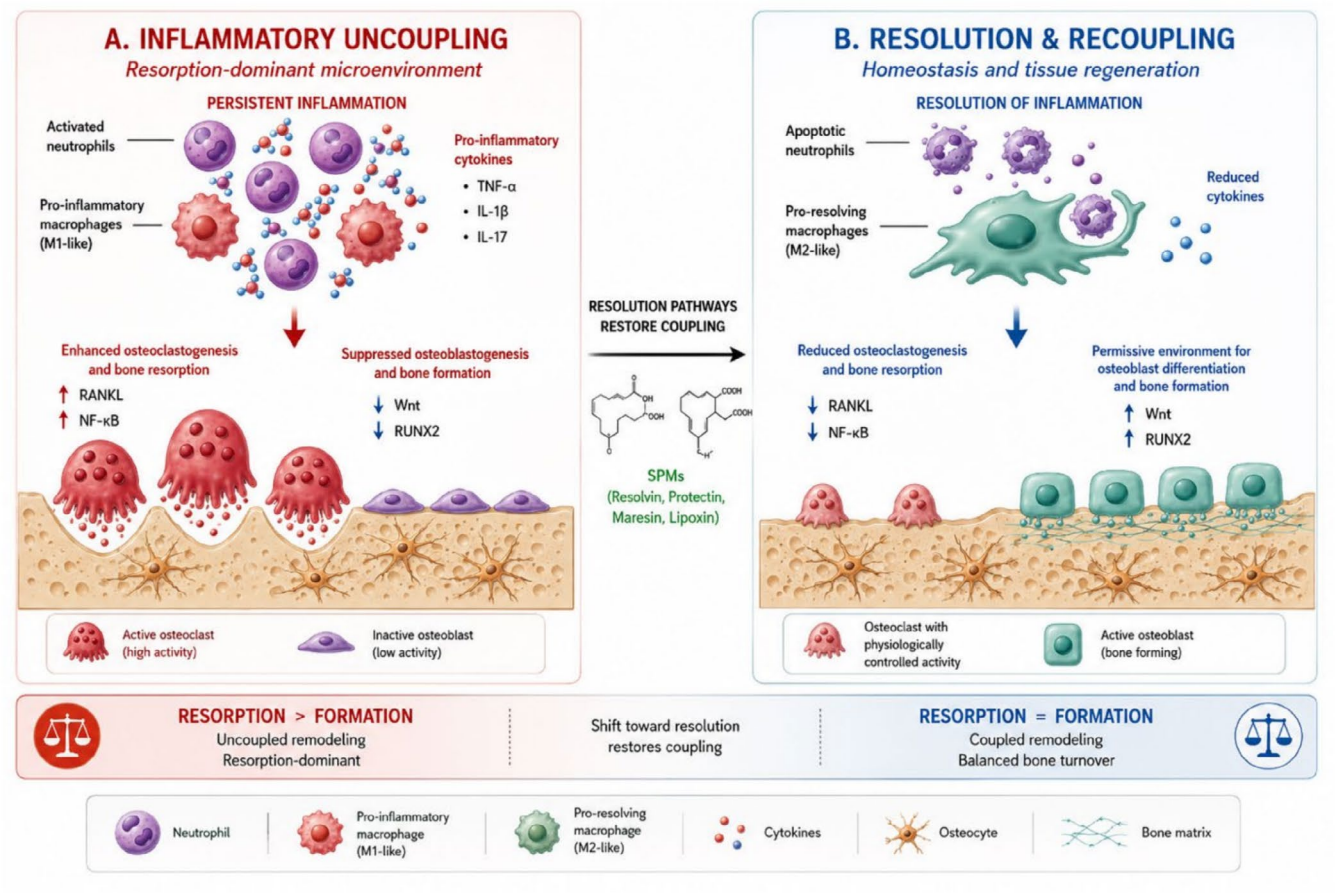
Resolution of inflammation restores osteoclast–osteoblast coupling in periodontitis. Chronic periodontal inflammation sustains a resorption‐dominant microenvironment characterized by persistent neutrophil activation, pro‐inflammatory macrophage polarization, and elevated cytokine production (e.g., TNF‐α, IL‐1β, IL‐17), which collectively drive NF‐κB‐dependent osteoclastogenesis and suppress osteoblast differentiation through inhibition of Wnt signaling and RUNX2 activity. This results in uncoupled bone remodeling, where resorption exceeds formation. In contrast, activation of endogenous resolution pathways by specialized pro‐resolving mediators (SPMs), including resolvins and lipoxins, limits neutrophil recruitment, promotes efferocytosis, and reprograms macrophages toward pro‐resolving phenotypes. These changes attenuate inflammatory signaling, reduce osteoclast activity, and relieve cytokine‐mediated suppression of osteoblast‐lineage cells, thereby restoring a permissive environment for bone formation. Collectively, resolution of inflammation re‐establishes physiological coupling between bone resorption and formation, enabling tissue regeneration and structural recovery. Created with BioRender.com (license no. CW29NC0NMG). (A. Inflammatory uncoupling), (B. resolution & recoupling).

An alternative and mechanistically distinct approach to restoring bone remodeling balance in periodontitis is to actively promote the resolution phase of inflammation. In physiological settings, inflammation is not only tightly regulated but also self‐limited, transitioning through a coordinated sequence that culminates in restoration of tissue homeostasis (Figure 6). This process is actively orchestrated by SPMs, a superfamily of endogenous lipid‐derived molecules that includes E‐series and D‐series resolvins, lipoxins, protectins, and maresins, generated from polyunsaturated fatty acids through enzymatic pathways involving lipoxygenases and cyclooxygenases [27, 28, 136, 258]. These mediators exert their actions through specific G‐protein‐coupled receptors, such as ChemR23/ERV1 for Resolvin E1 (RvE1) and ALX/FPR2 or GPR32 for Resolvin D1 (RvD1), initiating signaling cascades that terminate inflammation in a controlled manner while preserving essential antimicrobial defense mechanisms [176, 178]. In this context, periodontitis can be conceptualized not only as a state of excessive immune activation but also as conditions in which endogenous resolution pathways fail to be appropriately engaged, resulting in sustained cytokine production, persistent immune cell infiltration, and prolonged disruption of tissue homeostasis [27, 135, 137, 259].

The concept that resolution is an active biological program instead of a passive decay of inflammation was elegantly established through the work of Dr. Serhan, Dr. Van Dyke, and colleagues, demonstrating that lipid mediators such as lipoxins and resolvins function as endogenous “stop signals” that limit neutrophil recruitment, promote non‐phlogistic monocyte infiltration, and enhance macrophage‐mediated clearance of apoptotic cells [136]. These mechanisms are essential for preventing collateral tissue damage and enabling the transition from inflammation to repair. In periodontitis, failure of these pathways results in persistent neutrophil activation and sustained release of proteolytic enzymes and ROS, thereby amplifying tissue destruction and perpetuating a resorption‐dominant microenvironment.

Seminal experimental studies demonstrated that RvE1 exerts potent therapeutic effects in experimental models of periodontitis. In a well‐characterized rabbit model of ligature‐ and *Porphyromonas gingivalis* ‐induced disease, topical administration of RvE1 not only prevented disease progression but also reversed established periodontal inflammation and tissue destruction [259]. The study design included a 6‐week disease induction phase followed by therapeutic intervention. RvE1 treatment resulted in marked reduction of inflammatory infiltrate, decreased osteoclast activity, preservation of alveolar bone height, and restoration of periodontal architecture, providing the first direct evidence that resolution of inflammation can drive regeneration of previously lost tissues [259].

Beyond immunomodulation, RvE1 directly regulates bone cells: in murine osteoclast cultures it inhibits differentiation and resorptive activity through interference with RANKL‐induced NF‐κB via BLT1 and ChemR23, without affecting osteoclast survival [133].

Subsequent translational studies extended these findings to more complex and clinically relevant models. In ligature‐induced and surgically created periodontal defects, pro‐resolving nanomedicines incorporating lipoxin analogs or resolvins demonstrated the capacity to promote regeneration of both hard and soft tissues [27]. In a large animal model using miniature swine, localized delivery of benzo‐lipoxin A4 via nanoparticle‐based systems resulted in significant increases in new bone formation, reduced inflammatory infiltrate, and restoration of periodontal structure [27]. These studies highlight that effective regeneration in inflammatory bone diseases requires active engagement of resolution pathways.

At the cellular level, SPMs exert coordinated effects across innate and adaptive immunity that are directly relevant to inflammatory uncoupling. They limit excessive neutrophil recruitment, promote timely apoptosis, and enhance macrophage efferocytosis, facilitating clearance of inflammatory cells and debris. In parallel, they reprogram macrophages toward pro‐resolving phenotypes while reducing Th1 and Th17 responses, thereby attenuating cytokine networks that drive osteoclastogenesis [132, 135, 136, 137]. These actions result in suppression of key mediators such as TNF‐α, IL‐1β, and IL‐17A, ultimately reducing osteoclast precursor recruitment and differentiation. In addition, SPMs modulate the RANKL/OPG axis and inhibit NF‐κB signaling, thereby attenuating osteoclast activity and limiting bone resorption [133].

Equally important, resolution of inflammation alleviates cytokine‐mediated inhibition of osteoblast‐lineage cells, restoring the biological conditions required for bone formation. Although SPMs do not directly act as classical anabolic agents, their ability to suppress NF‐κB–dependent transcriptional repression and relieve Wnt pathway inhibition creates a permissive environment for osteoblast differentiation, matrix production, and mineralization. In this context, the regenerative outcomes observed in experimental models might be interpreted as reactivation of endogenous coupling mechanisms [27, 28, 134, 135, 136, 137].

Within the broader context of this manuscript, these findings provide a potential mechanistic link between immune dysregulation and impaired bone remodeling. Collectively, preclinical studies suggest that resolution biology may represent an important regulatory pathway in inflammatory bone loss. By attenuating inflammatory signaling and reducing osteoclastogenic stimuli, SPMs have demonstrated the capacity to favor a tissue environment more conducive to physiological bone remodeling. Although experimental evidence indicates beneficial effects on both inflammatory responses and tissue regeneration, the extent to which SPMs directly restore osteoclast–osteoblast coupling and re‐establish physiological remodeling dynamics in periodontitis remains incompletely understood. Consequently, SPMs should currently be viewed as promising host‐modulatory agents whose proposed effects on remodeling coupling and tissue regeneration warrant further mechanistic investigation and clinical validation.

##### 10.3.3.1 SPMs as Upstream Regulators of Osteoclast–Osteoblast Coupling Pathways

Beyond their direct anti‐inflammatory and anti‐osteoclastogenic actions, accumulating evidence suggests that SPMs may indirectly restore bone remodeling balance by modulating signaling pathways involved in osteoclast–osteoblast coupling [260, 261, 262]. In this context, SPMs might be viewed as upstream regulators qualified of re‐establishing the molecular environment required for physiological coupling.

Mechanistic support comes from studies made by El Kholy et al. [260], who demonstrated that in murine inflammatory osteolysis RvE1 acts directly on osteoblasts to reduce IL‐6‐induced RANKL production, shifting RANKL/OPG balance toward bone preservation, with transcriptomic modulation of NF‐κB, MAPK, and PI3K–AKT pathways that govern both osteoclastogenesis and osteoblast anabolic activity [260].

RvE1 also selectively impairs late‐stage osteoclast fusion by downregulating DC‐STAMP and inhibiting NFATc1 binding to its promoter, signaling through the BLT1 receptor to counteract leukotriene B4 and thereby fine‐tuning terminal osteoclastogenesis without abolishing physiological remodeling [262].

SPMs may also influence osteocyte‐mediated regulation of remodeling. Osteocytes are highly responsive to inflammatory and oxidative stress signals, which increase RANKL and sclerostin production while promoting osteocyte apoptosis. Because SPMs reduce neutrophil‐driven oxidative stress, suppress pro‐inflammatory cytokine signaling, and promote restoration of tissue homeostasis, they may indirectly preserve osteocyte viability and normalize osteocyte‐derived regulatory signals [132, 135, 136]. Preservation of osteocyte integrity is particularly relevant because osteocyte dysfunction contributes simultaneously to exaggerated osteoclastogenesis and impaired osteoblast activation, thereby amplifying remodeling uncoupling.

In vivo, Lee et al. [261] demonstrated that topical RvE1 both prevented and treated established ligature‐induced periodontitis in rats, reducing alveolar bone loss by approximately 30%–40%, partially regenerating lost alveolar bone, and decreasing osteoclast density. Transcriptomic analyses showed downregulation of inflammatory and bone‐resorption–related genes (e.g., Il1b, Ccl3, Cxcl1, Cxcl2, Mmp3, Mmp13, Acp5) reversing the disease‐associated expression signature toward a healthier profile [261]. Noteworthy, RvE1 also reshaped the subgingival microbiota despite lacking direct antimicrobial activity, supporting the concept that chronic inflammation itself sustains dysbiosis by altering the local ecological niche [261].

Although SPMs have demonstrated promising anti‐inflammatory, pro‐resolving, and tissue‐protective effects in multiple experimental models of periodontitis, the current evidence remains predominantly preclinical, and direct human clinical evidence is still limited (Tables 1 and 2). Existing studies support the concept that SPMs may favor restoration of a remodeling environment permissive to coupled bone formation by reducing inflammatory signaling and osteoclastogenic stimuli [27, 28, 258, 259, 261, 263]. However, their proposed effects on osteocyte‐derived coupling pathways, ephrinB2/EphB4 and semaphorin signaling, and Wnt‐regulatory networks remain largely inferred from broader resolution‐biology and skeletal‐remodeling literature instead of being directly demonstrated in periodontal tissues [132, 133, 135, 136, 137, 260, 262, 264]. Consequently, these mechanisms should presently be regarded as biologically plausible assumptions that warrant direct experimental validation in periodontitis.

#### 10.3.4 Immunomodulatory Therapies

Immunomodulatory therapies represent a targeted approach to controlling the host response in periodontitis by selectively reducing destructive inflammation while preserving essential immune defense mechanisms [20, 21]. Unlike broad anti‐inflammatory or immunosuppressive strategies, these approaches aim to recalibrate the immune response, limiting tissue damage without compromising the ability to control microbial challenge. This distinction is particularly important in periodontal tissues, where complete suppression of immunity is neither feasible nor desirable due to the constant presence of oral biofilms [218, 219, 220].

Targeting key inflammatory pathways can reduce tissue destruction; however, these approaches alone are insufficient to restore bone formation, highlighting the need for combined strategies that address both inflammation and regeneration [27, 107, 173, 234, 267]. Inhibition of these pathways has been shown to reduce inflammatory signaling, decrease matrix degradation, and limit disease progression in periodontal models. In parallel, host‐modulation approaches such as subantimicrobial‐dose doxycycline (SDD) have demonstrated the ability to reduce collagenase activity and matrix metalloproteinase‐mediated tissue destruction, leading to improvements in periodontal outcomes [218, 219, 220].

Previous studies [218, 219] investigating SDD as host‐modulatory therapy in periodontology provide important proof‐of‐concept that selective targeting of destructive inflammatory pathways can improve periodontal outcomes without exerting direct antimicrobial effects. Administered at 20 mg twice daily, SDD acts primarily through inhibition of MMPs, particularly collagenases and gelatinases involved in connective tissue degradation and bone resorption, while avoiding the antibacterial concentrations associated with conventional tetracycline therapy. Beyond its anti‐collagenolytic activity, experimental evidence suggests that doxycycline and related tetracycline derivatives may promote osteoblast function, collagen synthesis, and bone formation, supporting a broader role in preserving remodeling homeostasis [218, 219].

Clinical studies demonstrated that adjunctive SDD therapy significantly reduced gingival crevicular fluid collagenase activity and improved clinical attachment levels when combined with conventional periodontal therapy. Importantly, these benefits were associated with sustained suppression of host‐derived tissue‐destructive enzymes supporting the concept that modulation of the host response can complement mechanical biofilm control. Patients receiving the most effective SDD regimens exhibited minimal attachment loss over prolonged follow‐up, whereas placebo‐treated subjects showed continued disease progression [218].

Evidence that SDD may also influence alveolar bone remodeling emerged from a two‐year randomized clinical trial in postmenopausal osteopenic women with periodontitis [219]. Although no significant overall benefit was observed in the primary intention‐to‐treat analyses, subgroup analyses revealed reduced alveolar bone density loss in non‐smokers and at sites with probing depths ≥ 5 mm, together with a 29%–36% reduction in the odds of progressive alveolar bone height loss among women more than 5 years postmenopausal and among protocol‐adherent subjects. These findings suggest that host‐modulation strategies may be particularly effective in patients with active tissue breakdown and slower systemic bone turnover.

Equally important, long‐term microbiological analyses demonstrated that continuous SDD administration for up to 24 months did not alter the composition of the subgingival microbiota, promote overgrowth of opportunistic pathogens, or increase resistance to doxycycline or other commonly used antibiotics [220]. The percentage of doxycycline‐resistant isolates did not increase during treatment, and no evidence of clinically relevant cross‐resistance was observed. These observations confirmed the established long‐term safety of this approach.

Beyond cytokine inhibition, emerging approaches aim to rebalance immune cell populations and functions. Strategies that promote regulatory pathways, such as enhancing regulatory T cell activity or shifting macrophage polarization toward a reparative phenotype, have shown promise in experimental settings [21, 27, 108, 134, 172, 173, 174, 234]. Tregs exert protective effects by suppressing excessive Th1 and Th17 responses, reducing production of osteoclastogenic cytokines such as IL‐17A, TNF‐α, and RANKL, while simultaneously promoting anti‐inflammatory mediators including IL‐10 and TGF‐β. Through these mechanisms, Tregs help restrain osteoclast differentiation and activity while creating a microenvironment more permissive to osteoblast survival and bone formation. Conversely, impaired Treg function and expansion of pathogenic Th17 cells have been consistently associated with persistent inflammation, enhanced osteoclastogenesis, and remodeling uncoupling in periodontitis and other inflammatory skeletal diseases [21, 27, 108, 134, 172, 173, 174, 234].

Macrophage‐targeted therapies represent another interesting avenue for immunomodulation. Chronic periodontal inflammation is characterized by accumulation of classically activated, pro‐inflammatory macrophages that produce TNF‐α, IL‐1β, IL‐6, ROS, and other mediators that amplify tissue destruction and osteoclastogenesis. Experimental interventions that promote polarization toward reparative macrophage phenotypes have been shown to reduce inflammatory cytokine production, enhance efferocytosis, support extracellular matrix remodeling, and stimulate secretion of growth factors involved in tissue repair and angiogenesis [21, 134, 172]. Importantly, emerging evidence indicates that macrophage populations exist along a functional spectrum, and successful therapeutic strategies likely depend on restoring balanced macrophage responses instead of simply inducing a single phenotype. Such immune reprogramming may indirectly contribute to restoration of bone remodeling by reducing osteoclastogenic signals while enhancing conditions favorable to osteoblast differentiation and matrix deposition.

Similarly, modulation of intracellular signaling pathways involved in immune activation has attracted increasing interest as a means to attenuate chronic inflammation without fully suppressing host defense mechanisms. Among these pathways, NF‐κB occupies a central position because it integrates signals from microbial products, inflammatory cytokines, and damage‐associated molecular patterns to regulate expression of numerous mediators involved in leukocyte recruitment, cytokine production, matrix degradation, and osteoclast differentiation [27, 108]. Sustained NF‐κB activation contributes not only to persistent inflammation but also to excessive RANKL‐mediated osteoclastogenesis and inhibition of osteoblast function. Consequently, pharmacological strategies targeting NF‐κB signaling have demonstrated the capacity to reduce inflammatory bone loss in experimental periodontitis while preserving essential immune responsiveness [27, 108, 234]. Other intracellular pathways, including JAK/STAT, MAPK, PI3K/Akt, and NLRP3 inflammasome signaling, have also emerged as potential therapeutic targets due to their roles in coordinating inflammatory responses and regulating osteoimmune interactions. Collectively, these approaches seek to restore immune homeostasis and interrupt the self‐perpetuating inflammatory circuits that drive remodeling uncoupling.

Despite these advances, the direct impact of immunomodulatory therapies on bone remodeling remains limited when used in isolation. While reducing inflammation can decrease the stimuli that drive bone resorption, it does not necessarily restore the capacity for bone formation, particularly in tissues where osteoblast function is already impaired. In this context, immunomodulation primarily reduces the “catabolic pressure” exerted by the inflammatory environment but may not be sufficient to reinitiate the formation phase of remodeling.

For this reason, increasing attention has been directed toward combination strategies that integrate immunomodulation with approaches that promote bone formation or restore coupling mechanisms. By simultaneously dampening destructive inflammation and enhancing osteogenic potential, such combined therapies may achieve more complete restoration of tissue homeostasis. For example, coupling immunomodulatory interventions with agents that support Wnt signaling, osteoblast differentiation, or resolution of inflammation may create a more conducive microenvironment for regeneration. These considerations highlight the need to combine immunomodulation with complementary regenerative approaches tailored to disease stage.

### 10.4 Translational Challenges

Despite growing mechanistic insights, translating coupling‐focused strategies into clinical practice remains challenging. One major obstacle is the discrepancy between experimental models and human disease [235]. Most animal models of periodontitis are acute or subacute and do not fully reproduce the chronic, fluctuating course of human disease [1]. As a result, therapeutic effects observed in experimental settings may overestimate efficacy in patients with long‐standing inflammatory bone loss [235]. The timing of intervention is another critical determinant. Early treatment may allow recovery of coupling mechanisms before extensive osteocyte loss and structural damage have occurred. In advanced disease, however, the cellular machinery required for bone formation may already be irreversibly compromised, thereby limiting the effectiveness of anabolic therapies. Finally, site‐specific skeletal responses must be considered. Alveolar bone differs fundamentally from axial and appendicular bone in turnover rate, mechanical environment, and immune exposure. Therapeutic strategies that are effective in long bones may therefore not translate directly to the periodontium, emphasizing the need for localized delivery systems and site‐adapted dosing strategies [235].

## 11 Limitations

Some limitations should be considered when interpreting the concepts discussed in this review. First, as a narrative review, the present work does not follow a systematic methodology for study identification, selection, or quantitative synthesis. Although this approach allows integration of diverse fields including osteoimmunology, skeletal biology, aging, mechanobiology, and resolution pharmacology, it inherently introduces the possibility of selection bias and differential emphasis across topics.

Second, a substantial proportion of the evidence supporting inflammatory uncoupling derives from experimental animal models. While these models have provided invaluable mechanistic insights, important interspecies differences exist in immune responses, bone remodeling dynamics, osteocyte biology, and periodontal anatomy. Consequently, findings observed in rodents, rabbits, and other experimental systems cannot always be directly extrapolated to human periodontal disease.

Third, considerable heterogeneity exists among experimental models of periodontitis. Ligature‐induced, oral gavage, bacterial inoculation, genetic, aging‐associated, and systemic disease‐associated models each reproduce distinct aspects of periodontal pathology and may differ substantially in inflammatory intensity, microbial composition, rates of bone loss, and reparative responses. These differences complicate direct comparison between studies and may contribute to variability in reported mechanisms.

Fourth, the strength of evidence supporting individual components of the inflammatory uncoupling model is not uniform. Excessive osteoclastogenesis driven by RANKL, inflammatory cytokines, and osteoimmune interactions is strongly supported by both experimental and clinical evidence. In contrast, several mechanisms proposed to contribute to impaired bone formation, including osteocyte bystander signaling, Panx1/ATP‐mediated communication, ephrinB2/EphB4 coupling, semaphorin signaling, skeletal stem cell dysfunction, and certain mechanobiological pathways, remain supported primarily by evidence from broader skeletal biology, rheumatoid arthritis, osteoporosis, fracture healing, or other inflammatory bone diseases. Although these mechanisms are biologically plausible and consistent with current understanding of bone remodeling, direct periodontal validation remains limited.

Finally, human evidence supporting remodeling uncoupling remains less extensive than experimental evidence. Ethical and practical constraints limit direct investigation of bone formation dynamics, osteocyte behavior, and coupling mechanisms in human periodontal tissues. Consequently, many aspects of the proposed review remain inferential and require validation through longitudinal clinical studies, advanced imaging approaches, tissue analyses, and translational investigations.

Despite these limitations, the inflammatory uncoupling basis provides a useful conceptual model for integrating the diverse cellular and molecular processes involved in periodontal bone loss.

## 12 Concluding Remarks

The central premise of this review is that periodontitis represents more than a localized inflammatory bone disease; it provides a clinically accessible model of inflammatory uncoupling in which the normal coordination between bone resorption and bone formation becomes progressively disrupted. In this context, periodontitis illustrates how chronic inflammation disrupts coordinated bone remodeling, resulting in persistent imbalance between resorption and formation. Instead of a purely resorptive condition, periodontal bone loss reflects failure of the mechanisms that normally restore skeletal integrity following resorption. Positioning periodontitis within the broader context of inflammatory skeletal diseases highlights fundamental shared mechanisms, including RANKL‐driven osteoclastogenesis, Wnt pathway inhibition, and cytokine‐mediated suppression of osteoblast function. At the same time, the accessibility and localized nature of the periodontal lesion provide a distinctive opportunity to investigate these processes directly in humans. As such, periodontitis should be recognized not only as a prevalent oral disease but also as an informative in vivo model for studying inflammation‐driven remodeling failure and its systemic implications.

Importantly, the background presented herein should be viewed as an extension of earlier concepts proposing that inflammatory bone loss reflects disruption of physiological remodeling homeostasis [22]. Although many of the mechanisms discussed are supported by direct periodontal evidence [22, 54, 55, 64, 123, 150, 160], others are informed by advances in broader skeletal biology and should be considered priorities for future periodontal‐specific investigation [43, 45, 46, 50, 145, 191, 192, 201]. The novelty of the present synthesis lies not in redefining this principle but in integrating emerging evidence from multiple disciplines that have matured substantially in recent years, including osteocyte‐centered regulation of remodeling, SPM biology, age‐related alterations in skeletal responsiveness, and mechanobiological control of bone adaptation. Collectively, these advances provide a more comprehensive understanding of how chronic inflammation interferes with the coordinated processes required for maintenance and restoration of skeletal integrity.

From a therapeutic perspective, these insights support a shift away from strategies focused exclusively on inhibiting bone resorption toward approaches that restore physiological coupling. Interventions that combine control of inflammation, reactivation of anabolic pathways, and preservation of osteocyte function may offer more effective and durable restoration of bone homeostasis. Importantly, timing and disease stage are likely to be critical determinants of therapeutic success, as advanced lesions may involve irreversible alterations in cellular competence and tissue architecture.

Future research should prioritize longitudinal human studies integrating molecular, cellular, and imaging biomarkers to better define the dynamics of remodeling imbalance and recovery. Emphasis on osteocyte biology, skeletal progenitor function, and targeted therapeutic delivery will be essential to translate mechanistic insights into clinically effective strategies. Ultimately, advancing the field will require a more integrated view of bone biology in which immune regulation, mechanobiology, aging, and regenerative capacity are considered as interconnected determinants of remodeling outcomes. By leveraging the unique features of the periodontium, future studies have the potential not only to improve periodontal therapy but also to inform broader strategies for the prevention and treatment of inflammatory bone loss across the skeleton.

## Author Contributions

Conceptualization, Rafael Scaf de Molon, Sotirios Tetradis, Rolando Vernal, Fabio Renato Manzolli Leite and Thomas E. Van Dyke; Methodology, Rafael Scaf de Molon, Sotirios Tetradis, Rolando Vernal, Fabio Renato Manzolli Leite and Thomas E. Van Dyke; validation, Rafael Scaf de Molon, Sotirios Tetradis, Rolando Vernal, Fabio Renato Manzolli Leite and Thomas E. Van Dyke; formal analysis, Rafael Scaf de Molon, Sotirios Tetradis, Rolando Vernal, Fabio Renato Manzolli Leite and Thomas E. Van Dyke; investigation, Rafael Scaf de Molon, Sotirios Tetradis, Rolando Vernal, Fabio Renato Manzolli Leite and Thomas E. Van Dyke; data curation, Rafael Scaf de Molon, Sotirios Tetradis, Rolando Vernal, Fabio Renato Manzolli Leite and Thomas E. Van Dyke: writing – original draft preparation, Rafael Scaf de Molon, Sotirios Tetradis, Rolando Vernal, Fabio Renato Manzolli Leite and Thomas E. Van Dyke; writing – review and editing, Rafael Scaf de Molon, Sotirios Tetradis, Rolando Vernal, Fabio Renato Manzolli Leite and Thomas E. Van Dyke; visualization, Rafael Scaf de Molon, Sotirios Tetradis, Rolando Vernal, Fabio Renato Manzolli Leite and Thomas E. Van Dyke. All authors made substantial contributions to the study and were equally responsible for its design, execution, and content, and agreed to its submission for publication.

## Funding

Rafael Scaf de Molon is currently supported by a grant provided by the Sao Paulo Research Foundation—FAPESP. Grant #2023/15750‐7.

## Disclosure

This manuscript used NLP (ChatGPT) for proofreading an entirely human‐generated text, but no other use of AI was made.

## Conflicts of Interest

Dr. Van Dyke is inventor on several granted and pending licensed and unlicensed patents awarded to the ADA Forsyth Institute that are subject to consulting fees and royalty payments. He is a founder of Nocendra Inc. and AIAH LLC. The other authors declare they have no conflicts of interest related to the publication of this manuscript.

## Data Availability

Data sharing not applicable to this article as no datasets were generated or analysed during the current study.
